# Maternal body mass index and post‐term birth: a systematic review and meta‐analysis

**DOI:** 10.1111/obr.12489

**Published:** 2017-01-13

**Authors:** N. Heslehurst, R. Vieira, L. Hayes, L. Crowe, D. Jones, S. Robalino, E. Slack, J. Rankin

**Affiliations:** ^1^ Institute of Health and SocietyNewcastle UniversityNewcastle upon TyneUK

**Keywords:** BMI, gestational age, maternal, obesity

## Abstract

Post‐term birth is a preventable cause of perinatal mortality and severe morbidity. This review examined the association between maternal body mass index (BMI) and post‐term birth at ≥42 and ≥41 weeks' gestation. Five databases, reference lists and citations were searched from May to November 2015. Observational studies published in English since 1990 were included. Linear and nonlinear dose–response meta‐analyses were conducted by using random effects models. Sensitivity analyses assessed robustness of the results. Meta‐regression and sub‐group meta‐analyses explored heterogeneity. Obesity classes were defined as I (30.0–34.9 kg m^−2^), II (35.0–39.9 kg m^−2^) and III (≥40 kg m^−2^; IIIa 40.0–44.9 kg m^−2^, IIIb ≥ 45.0 kg m^−2^). Searches identified 16,375 results, and 39 studies met the inclusion criteria (*n* = 4,143,700 births). A nonlinear association between maternal BMI and births ≥42 weeks was identified; odds ratios and 95% confidence intervals for obesity classes I–IIIb were 1.42 (1.27–1.58), 1.55 (1.37–1.75), 1.65 (1.44–1.87) and 1.75 (1.50–2.04) respectively. BMI was linearly associated with births ≥41 weeks: odds ratio is 1.13 (95% confidence interval 1.05–1.21) for each 5‐unit increase in BMI. The strength of the association between BMI and post‐term birth increases with increasing BMI. Odds are greatest for births ≥42 weeks among class III obesity. Targeted interventions to prevent the adverse outcomes associated with post‐term birth should consider the difference in risk between obesity classes.

**Abbreviations**
BMI: body mass index
CI: confidence interval
IQR: inter quartile range
OR: odds ratio
RR: relative risk

## Introduction

Post‐term birth is a preventable cause of intra‐uterine death, stillbirth, neonatal and infant death 1, 2, 3, 4. Post‐term birth contributes to severe morbidities for the mother and child, including macrosomia, shoulder dystocia, birth injury, fourth degree perineal laceration, fetal compromise, antenatal and postpartum haemorrhage, fetal dysmaturity, labour >24 h and newborn respiratory distress syndrome 1, 5, 6, 7. There is emerging evidence that primiparous women who deliver post term have an increased risk of developing type 2 diabetes in later life 8. Costly obstetric and neonatal interventions associated with post‐term birth include caesarean section, induction of labour, operative vaginal delivery, close fetal monitoring beyond term, ventilator use and neonatal intensive care admission 1, 7, 9. The risks associated with post‐term birth have historically been under‐estimated due to self‐reported assessment of gestational age relying on last menstrual period. This self‐report assessment over‐estimates post‐term prevalence, resulting in an underestimate of the risks of ‘true’ post‐term birth due to lower‐risk ‘term’ births being misclassified as post‐term 1, 4, 6. Current widespread use of ultrasound scan technology provides a more accurate estimation of gestational age 10 and allows exploration of the ‘true’ post‐term risks.

Maternal obesity (i.e. pre‐pregnancy body mass index [BMI] ≥30 kg m^−2^) impacts on daily clinical practice due to the international rise in its prevalence and the complexity of its comorbidities. Maternal obesity is a complex condition strongly associated with socio‐economic status and ethnicity inequalities, 11, 12 making it a public health priority in addition to being a priority area for clinical practice. For example, socio‐economic status varies between obesity classes, and pregnant women in the highest obesity class (class III, BMI ≥40 kg m^−2^) are significantly more likely to reside in deprived locations (odds ratio [OR] 4.7, 95% confidence interval [CI] 3.2–6.9) compared with women in obesity class I (BMI 30.0–34.9 kg m^−2^; OR 2.2, 95% CI 2.1–2.3) 11. Disparities are also seen with maternal employment status. Pregnant women with a BMI in class I are more likely to be employed, while those in class III are more likely to be unemployed 11. Obesity‐associated adverse pregnancy outcomes for the mother and child include poorer mental health 13, gestational diabetes, 14 congenital anomalies 15 and perinatal mortality 2, 16. Pre‐pregnancy weight is the most significant modifiable risk factor for stillbirth, with up to 100% increased risk for women with obesity 22. There is increasing evidence that maternal BMI influences gestational age at delivery. Robust meta‐analysis data demonstrate the relationship between BMI and pre‐term birth 17, 18. Despite published studies exploring the association between maternal BMI and post‐term birth 19, 20, 21, there is a lack of robust evidence from meta‐analyses.

Both maternal obesity and post‐term birth are preventable, and therefore warrant intervention to prevent associated adverse outcomes. Challenges to investigating maternal obesity and post‐term birth include interventions to expedite birth, such as induction of labour and caesarean section, interrupting the natural gestation trajectory. There are differences in the definitions used to classify post‐term in existing literature, including pregnancies progressing beyond 40, 41 or 42 weeks of gestation 4, 6. Although there is evidence of significantly increased risks for each definition of post‐term beyond 40 weeks 22, the greatest risk is among the gestations >42 weeks for most adverse outcomes 9. The terminologies post‐term and prolonged pregnancy are also used interchangeably to describe gestational ages beyond term 4.

Investigation of the association between maternal obesity and post‐term birth adds additional complexity. Maternal obesity is associated with a significantly increased risk of developing the comorbidities which lead to early intervention and disrupts the natural pregnancy trajectory, including gestational diabetes and preeclampsia 14, 22. In addition, the BMI definitions used to categorize maternal weight status are used inconsistently, contributing to difficulty of interpretation when making direct comparisons of studies. The World Health Organization (WHO) criteria for categorizing BMI are <18.5 kg m^−2^ (underweight), 18.5–24.9 kg m^−2^ (recommended weight), 25.0–29.9 kg m^−2^ (overweight) and ≥30.0 kg m^−2^ (obese), with further obesity sub‐classes of class I 30.0–34.9 kg m^−2^, class II 35.0–39.9 kg m^−2^ and class III ≥40 kg m^−2^ obesity 23. For Asian populations, the BMI criteria are reduced (recommended weight 18.5–23 kg m^−2^, overweight 23–27.5 kg m^−2^ and obese >27.5 kg m^−2^) due to increased risk of metabolic diseases at a lower BMI 24. However, the Asian‐specific definitions for weight status are not consistently adopted internationally in research or clinical guidelines.

Overcoming the methodological challenges to establish the relationship between BMI and post‐term birth is important to inform strategies for preventing associated adverse outcomes, such as perinatal mortality and severe morbidity. Additionally, identification of the dose–response association would inform preconception and antenatal healthcare planning, practice and guidelines such as risk communication and shared decision‐making for intervention options for targeted groups of women based on BMI. This systematic review and meta‐analyses aimed to establish the strength of the association between maternal obesity and post‐term birth. It specifically investigated the dose–response association between BMI and post‐term birth, taking into consideration the methodological challenges, confounding and sources of heterogeneity in the existing research.

## Methods

Search strategies for systematic reviews of observational epidemiological studies require multiple components as database searches alone have been shown to only identify up to half of the relevant literature 25. Systematic exclusion of studies through following an inadequate search strategy increases the risk of publication bias. Therefore, a six‐stage search strategy was followed in an attempt to limit the effect of publication bias arising from searching literature databases alone.

**Figure 1:**
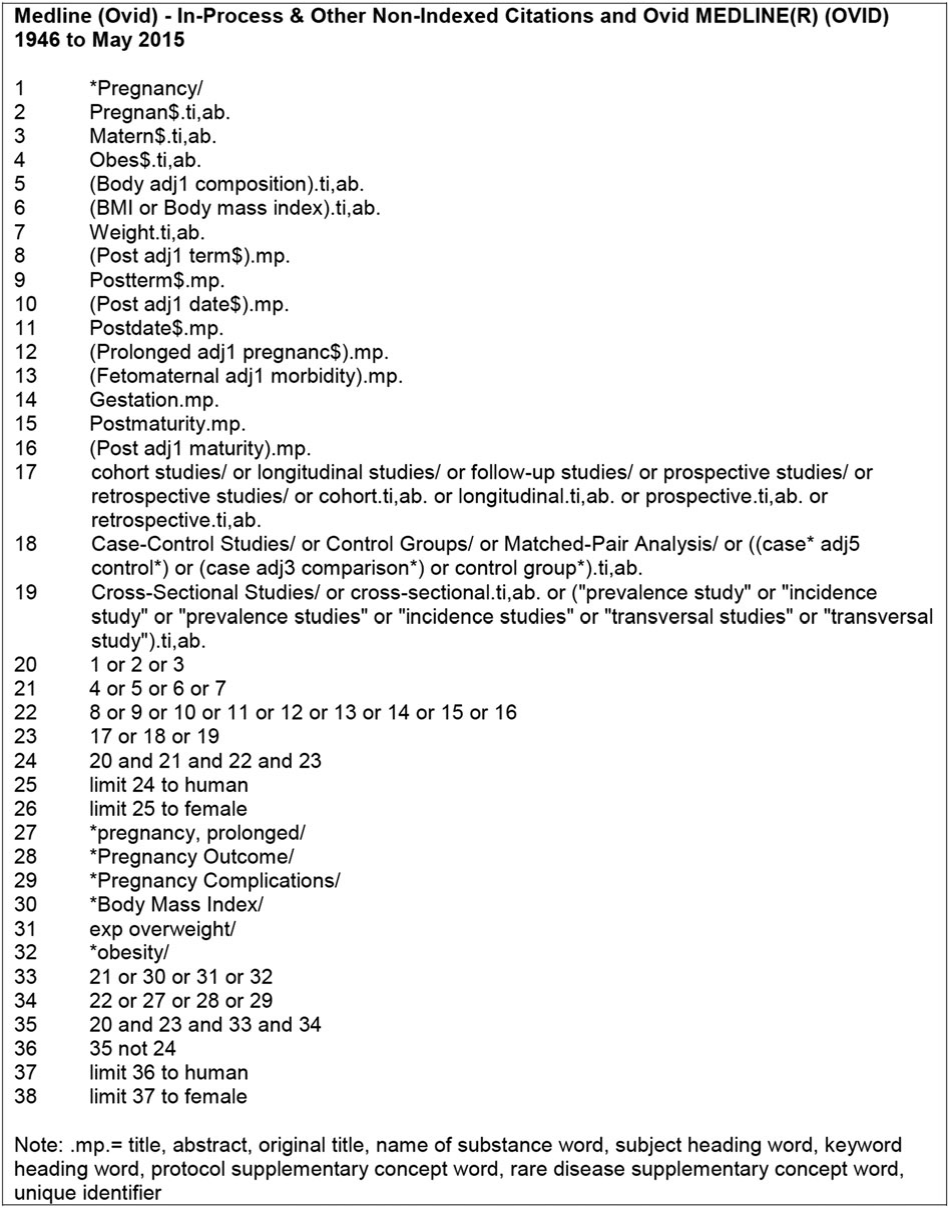
MEDLINE database search.

Stage 1: Databases were searched by using keywords and study filters for non‐randomized control trial studies. Restrictions to human studies were included. Search terms and subject headings were developed for MEDLINE (Fig. 1) and translated across four additional databases: British Nursing Index, Cumulative Index of Nursing and Allied Health, Embase and PsycInfo (Fig. S1).
Stage 2: The reference lists of all included studies, and all related systematic reviews identified in stage 1, were hand searched.
Stage 3: Citation searches for all included studies were performed by using Google Scholar citation function.
Stage 4: Authors of relevant published abstracts were contacted to identify if there had been subsequent full publication of studies.
Stage 5: Any additional studies identified in stages 2–4 were subject to further reference list and citation searching. Stages 2–5 continued until no further new studies were identified.
Stage 6: Authors of included studies were contacted for additional data when required for inclusion in the meta‐analyses.

Inclusion criteria were peer‐reviewed full studies (i.e. not abstracts, editorials, etc.), published in the English language since 1 January 1990. Studies had to report both the exposure variable (maternal weight status) and the outcome variable (post‐term birth). The six‐stage search strategy was carried out between May and November 2015. Screening titles, abstracts and full papers for inclusion in the review was carried out by two researchers independently. Data extraction and quality assessment were also carried out independently by two researchers by using a standardized protocol for data extraction (Table S1) and the Newcastle–Ottawa scale for cohort studies for quality assessment (Fig S2). Independent extractions and assessments were combined and agreed. A third researcher was available for any disagreements (not required).

In circumstances where there were missing or unclear definitions for the exposure or outcome variables, or missing frequency data, the authors were contacted for clarification. If the authors did not respond to the request for further information after follow‐up email requests, or if the authors could not be contacted for any reason, then assumptions about the definitions were made based on the information provided in the papers. For example, if the study described that they had compared post‐term (defined as ≥42 weeks) and pre‐term (defined as <37 weeks) with term (undefined), then the assumption was made that term was defined as the gestational age between the reported post‐ and pre‐term (37 to 41 + 6 weeks). Alternative methods of making assumptions included searching for definitions in papers that the authors had referenced in relation to gestational age or BMI and searching for any publications by the same authors on a similar topic where they had defined the variables. In the absence of any information to inform our assumptions following these methods, the terminology used by the authors was used to define the exposure and outcome variables. For example, if the authors used the term ‘normal BMI’, then the WHO criterion of 18.5–24.9 kg m^−2^ was assumed.

For the purposes of this systematic review, we categorized post‐term birth into two outcome variables which were analysed separately. The primary outcome was post‐term birth ≥42 weeks of gestation as this gestation incurs the greatest risk associated with post‐term birth, and the secondary outcome was post‐term birth ≥41 weeks of gestation as this gestation also has increased risk but to a lesser extent than 42 weeks. Dose–response meta‐analyses were conducted to investigate the association between maternal BMI and both outcomes. The study‐specific linear trends (ORs for continuous BMI assuming linearity) were derived by using the method by Greenland and Longnecker 26. This method requires the ORs with CIs for at least two exposure categories (including the reference group) and the number of cases and participants in each exposure category. If the adjusted ORs and CIs were not available, then the respective unadjusted parameters were derived from the data and used in the meta‐analysis. To assess the effect of including adjusted and unadjusted ORs in the meta‐analysis, subgroup meta‐analyses were performed with the studies that reported both adjusted and unadjusted ORs (or provided data to enable unadjusted ORs to be calculated), and the statistical significance and direction of the associations were compared. For each exposure category, the midpoint was calculated as the average of the lower and upper bound, and the respective OR was assigned to each midpoint. As the BMI midpoint was required for these analyses, upper and lower cut‐offs were applied to open‐ended BMI categories in increments of 5 BMI units (e.g. for BMI <18.5 kg m^−2^, a 5 BMI unit lower limit of 13.5 kg m^−2^ was applied; the respective midpoint was 16 kg m^−2^). The regression coefficient for a change of 5 BMI units (log OR_5BMI_) is a function of the coefficient estimated when assuming a change of 1 BMI unit (log OR_BMI_), such that log OR_5BMI_ = 5 × log OR_BMI_. The summary ORs were calculated by using the random effects model by DerSimonian and Laird 27.

A two‐stage, random‐effects, nonlinear dose–response meta‐analysis 28, 29 was also conducted to assess potential nonlinear associations, using cubic spline regression to model maternal BMI. The first stage involved fitting a cubic spline model with two spline transformations, accounting for the correlation within each set of published ORs. The two regression coefficients were combined, and the variance/covariance matrices were estimated for each study by using a random‐effects meta‐analysis. Nonlinearity was assessed by testing that the coefficient of the second spline was equal to zero 30. This method required ORs with CIs to be available for at least three exposure (BMI) categories, as when only two categories are reported (e.g. recommended and obese BMIs), information on how the outcome behaves between the two categories is not available, and nonlinearity cannot be assessed. Therefore, studies reporting data for only two BMI categories were excluded from the nonlinear analyses.

Publication bias was tested for using Eggers test 31. A two‐sided *p‐*value <0.05 was considered statistically significant. Sensitivity analyses were performed by systematically excluding one study at a time from the meta‐analysis. Meta‐regression and sub‐group meta‐analyses were carried out to explore factors identified *a priori* as being potentially important sources of heterogeneity. *A priori* clinical factors were the method of assessment of the exposure and outcome variables (maternal weight and gestational age at delivery) and consideration of the clinical confounders which impact on gestational age at delivery (induction of labour, elective caesarean section, parity, gestational diabetes, hypertension and pre‐eclampsia). No studies were excluded from the overall meta‐analysis based on methodological factors such as quality. However, methodological factors, including quality as well as study size, geography, age and duration of the data included, study design (e.g. retrospective or prospective, number of exposure categories and adjusted data) and how studies were identified for inclusion in the review were explored by meta‐regression and sub‐group meta‐analysis. Heterogeneity among studies was evaluated by using the *I* ^2^ statistic 32 with a threshold of >75% representing considerable heterogeneity 33. The statistical analyses were conducted by using stata version 13.1. Studies which met the inclusion criteria but did not present data suitable for inclusion in the meta‐analyses are summarized narratively. The systematic review was registered on the PROSPERO database (reference CRD42015014164).

## Results

**Figure 2:**
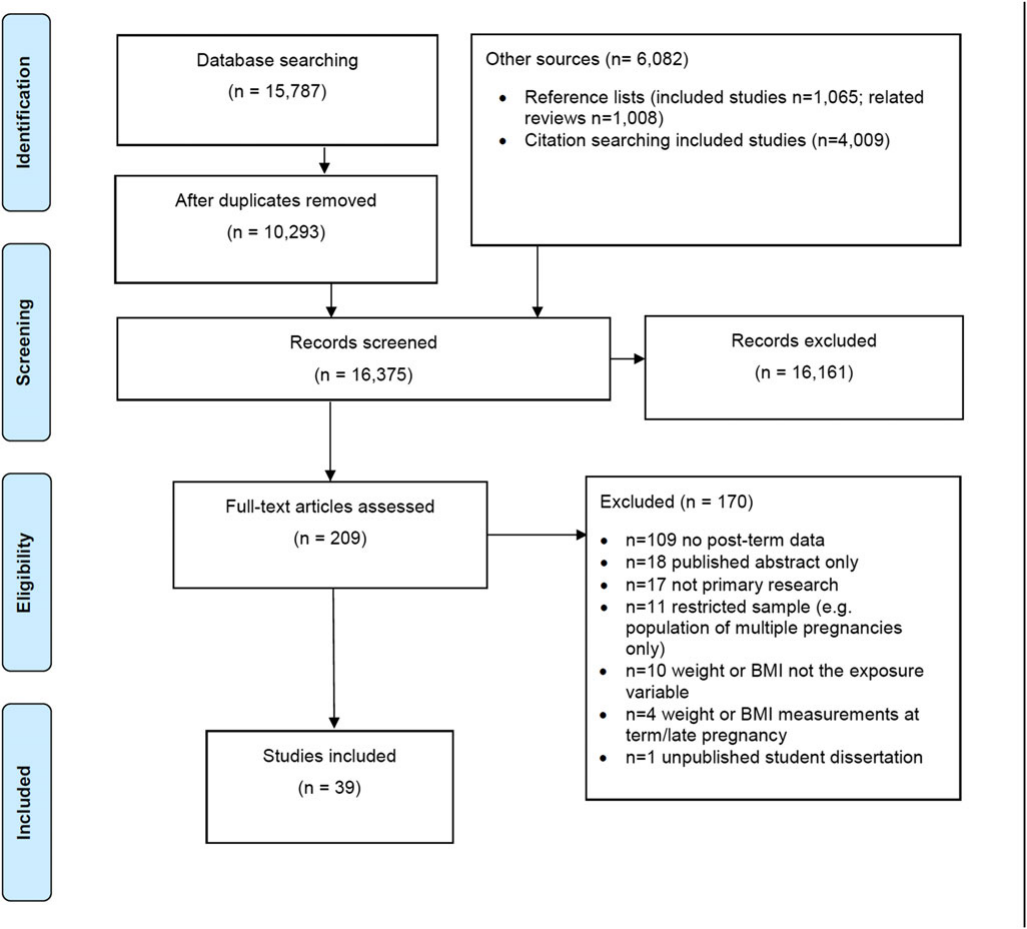
PRISMA flowchart of searches, screening and inclusion and exclusion of studies. [Colour figure can be viewed at wileyonlinelibrary.com]

Searches identified 16,375 studies, of which 39 met the inclusion criteria, giving a total population of 4,143,700 births (Fig. 2, Table S2 for detailed information on screening). Of the included studies, 24 (62%) were identified through database searches and 15 (38%) by searching reference lists and citations. Contacting authors of published abstracts did not identify any additional eligible studies. Of the 39 included studies, 26 reported data for post‐term birth ≥42 weeks, and 14 reported ≥41 weeks (see Table 1 for summary of included studies and Table S3 for additional detail). Some studies provided data for both definitions of post‐term (Table 1). Twenty studies were from Europe, five each from the USA and Middle East, four from Asia, three from Canada and one each from South Africa and Australia. Most studies were published between 2005 and 2014 (*n* = 33). Additional information was requested from the authors on definitions used (e.g. BMI or gestational age categories) or frequencies (e.g. number of cases or controls) for 34 studies (Table S4). The quality of studies ranged from a score of one to eight, with a median quality score of four (Tables 1 and S4 for detailed quality assessment results).

**Table 1:** Summary of included studies

Author, publication year, country	Study period	Gestational age categories (weeks)	BMI (kg m^−2^) or weight categories	Crude analysis (OR and 95% CI unless specified)	Adjusted analysis (OR and 95% CI unless specified)	Quality score (out of 8)
Abenhaim *et al*. 2007,^41^ Canada	04/1987–03/1997	>37–42* >42	20–24.9† <19.9 25–29.9 30–39.9 >40	Not reported	1(1) 1.07 (0.86–1.33) 1.13 (0.89–1.45) 0.84 (0.55–1.28) 0.76 (0.19–3.10)	3
Al‐Rayyan *et al*. 2010,^42^ Jordan	01/1990–12/2000	37–41* >42	<30† ≥30.0	Not reported	Not reported	2
Arora *et al*. 2013,^48^ Thailand	02/2011–08/2012	37–41* 42	18.5–24.9^†^ <18.5 25–29.9 ≥30	Not reported	Not reported	3
Arrowsmith *et al*. 2011,^58^ UK	01/2004–12/2008	37–41^+2^ * 41^+3^	20–24.9† <19.9 25–29.9 30–34.9 35–39.9 >40	Not reported	1(1) 0.75 (0.66–0.85) 1.24 (1.14–1.34) 1.52 (1.37–1.70) 1.75 (1.48–2.07) 2.27 (1.78–2.86)	8
Basu *et al*. 2010,^61^ South Africa	02/2006 and 09/2006	37–41* >41	18.5–24.9† 25–29.9 30–39.9 >40	Not reported	Not reported	3
Bhattacharya 2007,^63^ UK	1976–2005	37–41* >41	20–24.9† <19.9 25–29.9 30–34.9 >35	1 (1) 0.7 (0.6–0.8) 1.2 (1.1–1.3) 1.4 (1.1–1.6) 0.8 (0.4–1.7)	1 (1) 0.9 (0.7–1.1) 0.9 (0.8–1.1) 0.9 (0.7–1.1) 0.8 (0.4–1.8)	5
Briese *et al*. 2011,^38^ Germany	1998–2000	Not reported	18.5–24.9 ≥30	Not reported	1 (1) 1.45 (1.38–1.52)	4
Caughey *et al*. 2009,^21^ USA	01/1995–12/1999	37–<41* ≥41 37–<42* ≥42	Not obese† Obese (BMI not defined)	Not reported	1 (1) 1.29 (1.18, 1.40) 1 (1) 1.20 (0.99, 1.46)	4
Cedergren 2004,^49^ Sweden	01/1992–12/2001	37–41^+6^ * ≥42	19.8–26† 29.1–35 35.1–40 >40	Not reported	1 (1) 1.37 (1.33–1.41) 1.49 (1.40–1.58) 1.80 (1.62–2.01)	5
Denison *et al*. 2008,^39^ Sweden	1998–2002	37–41^+6^ * ≥42	20–25† <20 25− < 30 30− < 35 ≥35	Term median BMI 22.9 (IQR 21.0−25.3); post‐term median BMI 23.4 (IQR 21.5–26.0) *p* < 0.0001	Not reported	5
El‐Gilany and Hammad 2010,^50^ Saudi Arabia	01/2007–12/2007	37–42* >42	18.5–24.9† <18.5 25–29.9 ≥30	RR (95% CI) 1 (1) 2.3 (0.4–12.3) 2.0 (0.6–7.1) 3.7 (1.2–11.6)	Not reported	3
Halloran *et al*. 2012,^19^ USA	2000–2006	37–40* =41 =42	18.5–24.9† <18.5 25–29.9 ≥30	Not reported	Not reported	5
Johnson *et al*. 1992,^51^ USA	01/1987–12/1989	38–42* >42	<19.8† 19.8–26 27–29 >29	1 (1) 1.22 (0.89–1.66) 1.58 (1.03–2.4) 1.49 (1.01–2.2)	Not reported	5
Khashan and Kenny 2009,^20^ UK	01/2004–12/2006	Not reported* ≥41	18.5–24.9† <18.5 25–29.9 30–40 >40	1 (1) 0.79 (0.65–0.96) 1.13 (1.06–1.21) 1.28 (1.19–1.38) 1.17 (0.95–1.43)	1 (1) 0.81 (0.67–0.99) 1.17 (1.09–1.25) 1.35 (1.25–1.45) 1.24 (1.02–1.52)	5
Kistka *et al*. 2007,^40^ USA	1989–1997	37–41^+6^ * ≥42	Reference not defined† <20 >35	1 (1) 0.90 (0.88–0.93) 1.25 (1.19–1.32)	1 (1) 0.85 (0.82–0.87) 1.23 (1.16–1.29)	4
Kitiyodom and Tongswatwong 2008,^67^ Thailand	10/2004–09/2006	Reference not defined† Post‐term not defined	20–24.9† >25	1 (1) 1.7 (1.19–2.44)	Not reported	3
Knight *et al*. 2010,^68^ UK	09/2007–08/2008	Reference not defined† >42	<50† ≥50	1 (1) 1.31 (0.76–2.25)	1 (1) 1.35 (0.77–2.37)	4
Konje *et al*. 1993,^72^ UK	01/1989–06/1990	37–42* >42	17–24† 30.4–53.0	Not reported	Not reported	4
Leung *et al*. 2008,^34^ Hong Kong	01/1995–12/2005	37–40^+6^ * ≥41	18.5–<23† <18.5 ≥23–<25 ≥25–<27.5 ≥27.5–<30 ≥30	Not reported	1 (1) 0.84 (0.74–0.95) 1.06 (0.97–1.17) 1.21 (1.08–1.36) 1.25 (1.05–1.48) 1.34 (1.09–1.66)	4
Lumme *et al*. 1995,^35^ Finland	07/1985–06/1986	37–41* >41	19–24.9† <19 25–29.9 ≥30	Not reported	1 (1) 1.0 (0.7–1.4) 1.6 (1.2–2.1) 1.1 (0.6–1.9)	4
Mancuso *et al*. 1991,^36^ Italy	Not reported	38–41* >42	15.2–26.6† >30	Not reported	Not reported	1
Manzanares *et al*. 2012,^37^ Spain	2007–2009	37–41^+2^ * >41^+3^	18.5–25† <18.5 >35	Not reported	1 (1) 0.81 (0.35–1.91) 0.72 (0.34–1.55)	4
Morgan *et al*. 2014,^43^ UK	11/2010–02/2013	Reference not defined† = 42	18.5–24.9† >25	1 (1) 2.18 (0.99–4.84)	Not reported	4
Navid *et al*. 2013,^69^ Pakistan	05/2011–07/2012	37–40* >40	18–24.9† 25–35	Not reported	Not reported	2
Nohr *et al*. 2009,^70^ Denmark	1996–2002	37–41* >41	15–33.3† 32.6–<35 35–<37.5 ≥37.5	Not reported	1 (1) 1.3 (1.1–1.5) 1.5 (1.3–1.8) 1.4 (1.2–1.7)	4
Olesen *et al*. 2006,^65^ Denmark	1996–2004	37–41+6* ≥42	20–24† <20 25–29 30–34 ≥35	1 0.87 1.23 1.35 1.48 95% CI not reported	1 (1) 0.87 (0.80–0.94) 1.24 (1.15–1.34) 1.37 (1.22–1.54) 1.52 (1.28–1.82)	3
Raatikainen *et al*. 2006,^53^ Finland	01/1989–12/2001	Reference not defined† >42	≤25† 26–29 ≥30	Not reported	Not reported	5
Robinson *et al*. 2005,^37^ Canada	01/1988–12/1992	Reference not defined† > 41	55–75 Kg† ≥90–120 Kg >120 Kg	1 (1) 1.10 (1.01–1.20) 0.91 (0.67–1.23)	1 (1) 1.18 (1.08–1.28) 0.99 (0.74–1.34)	4
Rode *et al*. 2005,^54^ Denmark	1998–2001	37–42* >42	<25† 25–29.9 ≥30	Not reported	1 (1) 1.4 (1.2–1.7) 1.4 (1.1–1.9)	5
Roos *et al*. 2010,^55^ Sweden	01/1992–12/2006	37–41^+6^ * ≥ 42	20–24.9^†^ <20 25–29.9 ≥30	Not reported	1 (1) 0.74 (0.72–0.76) 1.31 (1.29–1.33) 1.63 (1.59–1.67)	8
Schrauwers and Dekker 2009,^62^ Australia	01/2006–06/2006	37–41* >41	19.1–25† 25.1–30 30.1–40 >40	Not reported	Not reported	2
Scott‐Pillai *et al*. 2013,^59^ UK	2004–2011	Reference not defined† > 41	18.5–24.99† <18.50 25–29.99 30–34.99 35–39.99 ≥40	Not reported	1 (1) 0.5 (0.2–1.0) 0.9 (0.7–1.1) 0.8 (0.5–1.1) 0.9 (0.5–1.6) 0.8 (0.4–1.7)	7
Sharief and Tarik 2000,^36^ Iraq	12/1997–08/1998	Reference not defined†, post‐term not defined	≤90 Kg >90 Kg	Not reported	Not reported	3
Stotland *et al*. 2007,^56^ USA	1990–2001	37–<41* ≥41	19.8–26† <19.8 26.1–29 >29	Not reported	1 (1) 0.83 (0.72–0.95) 1.29 (1.10–1.52) 1.81 (1.50–2.18)	6
		37–<42* ≥42	19.8–26† <19.8 26.1–29 >29		1 (1) 0.78 (0.60–1.01) 1.51 (1.15–1.97) 1.69 (1.23–2.31)	
Usha Kiran *et al*. 2005,^44^ UK	1990–1999	37–41* >41	20–30† >30	1 (1) 1.4 (1.2–1.7)	Not reported	4
Vaswani and Balachandran 2013,^60^ United Arab Emirates	12/2010–10/2011	37–41* >41	18.5–24.9† 25–29.9 30–34.9 35–39.9 ≥40	Not reported	1 (1) 1.54 (0.89–2.65) 1.69 (0.96–2.98) 1.78 (0.93–3.42) 2.99 (1.35–6.65)	4
Vinturache *et al*. 2014,^57^ Canada	05/2008–12/2010	37–41^+6^ * ≥42	18.5–24.99† 25–29.99 ≥30	Not reported	Not reported	5
Voigt *et al*. 2008,^71^ Germany	1998–2000	Term, not defined†; Post‐term, not defined	18.5–24.99† 40–44.99 ≥45	Not reported	Not reported	2
Yazdani *et al*. 2006,^66^ Iran	2008–2009	Term, not defined†; Post‐term, not defined	20–24.9† ≤19.9 25–29.9 30–34.9 >35	Not reported	Not reported	2

† Reference group for BMI.

* Reference group for gestational age.

Abbreviations: BMI, body mass index; CI, confidence interval; IQR, inter quartile range; OR, odds ratio; RR, relative risk.

There was negligible influence of using unadjusted or adjusted ORs in the analysis of either post‐term birth categories with a difference in OR of 0.03 when comparing adjusted and unadjusted data from the same studies (Fig. S3). Therefore, adjusted ORs were used when reported and unadjusted ORs in the absence of adjusted data. One study used the Asian‐specific BMI reference criteria 34. These data were transformed to represent the general population BMI criteria with no influence on the overall effect size (Fig. S4).

Nineteen studies reported data that could be pooled for meta‐analysis of post‐term birth ≥42 weeks, and 11 studies reported data for post‐term birth ≥41 weeks (some studies reported multiple outcomes). Data from 10 studies could not be included in the meta‐analysis, and a narrative summary is provided for the results of these studies.

### Meta‐analyses of post‐term birth ≥42 weeks of gestation

**Figure 3:**
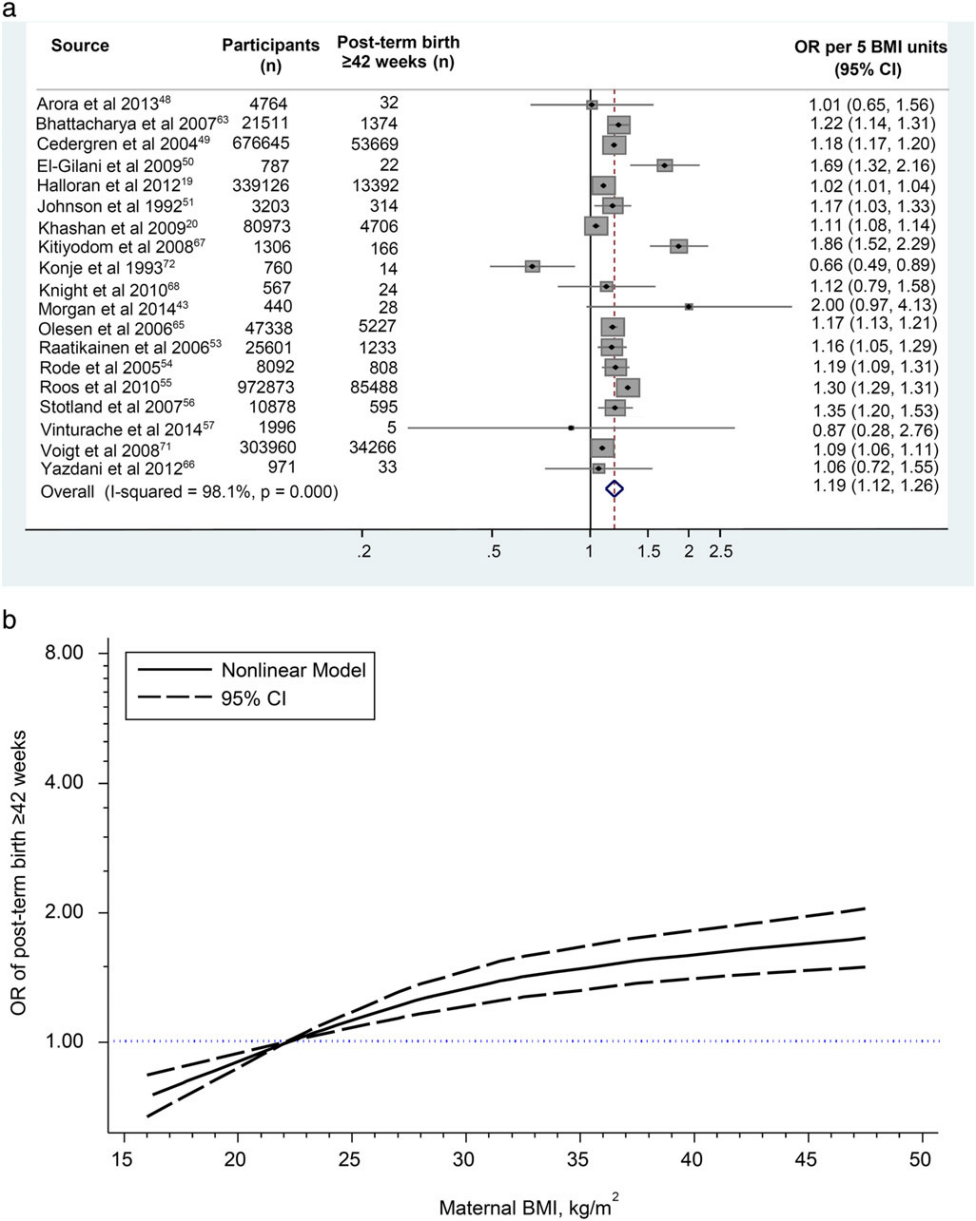
Linear and nonlinear dose–response association between maternal body mass index and post‐term birth ≥42 weeks: (3a) linear odds ratio per 5 maternal body mass index units. The squares and lines through the squares represent the study‐specific odds ratios and 95% confidence intervals. The dimension of the square is proportional to the weight of the study in the meta‐analysis. The diamond represents the summary odds ratio. (3b) nonlinear dose–response analysis. [Colour figure can be viewed at wileyonlinelibrary.com]

The 19 studies with data for ≥42‐week meta‐analysis included 201,396 cases among 2,501,803 pregnancies (8.1% incidence). In the dose–response analysis, the OR for each 5 unit increase or decrease in BMI compared with the reference BMI midpoint (22 kg m^−2^) was 1.19 (95% CI 1.12–1.26; heterogeneity *I* ^*2*^ = 98.1%, *p* < 0.001; Fig. 3a). There was evidence of a nonlinear association (*p* = 0.002, Table S6a and Fig. 3b) with a statistically significant decrease in odds of births ≥42 weeks for underweight BMI compared with the reference group and an increase for overweight and obese BMIs (Table 2). The odds of birth ≥42 weeks increased within obesity classes, with 42%, 55%, 65% and 75% increased odds for BMI classes I, II, IIIa and IIIb respectively (Table 2). There was no evidence of publication bias in the analyses of births ≥42 weeks (*p* = 0.60, Table S7).

**Table 2:** Odds ratios from linear and nonlinear dose‐response analyses for maternal BMI and post‐term birth

		BMI Class (Midpoint BMI, kg/m^2^)						
Post‐term category	Model	Underweight (17.5)	Reference BMI (22.5)	Overweight (27.5)	Obese I (32.5)	Obese II (37.5)	Obese IIIa (42.5)	Obese IIIb (47.5)
≥42 weeks	Linear, OR (95% CI)	0.84 (0.76,0.94)	1	1.19 (1.12,1.27)	1.38 (1.31,1.46)	1.57 (1.50,1.64)	1.76 (1.69,1.83)	1.95 (1.88,2.02)
≥42 weeks	Nonlinear, OR (95% CI)	0.81 (0.74,0.88)	1	1.24 (1.15,1.34)	1.42 (1.27,1.58)	1.55 (1.37,1.75)	1.65 (1.44,1.87)	1.75 (1.50,2.04)
≥41 weeks	Linear, OR (95% CI)	0.88 (0.83,0.95)	1	1.13 (1.05,1.21)	1.26 (1.18,1.34)	1.39 (1.31,1.47)	1.52 (1.44,1.54)	ND
≥41 weeks	Nonlinear, OR (95% CI)	0.91 (0.85,0.97)	1	1.11 (1.04,1.20)	1.22 (1.07,1.39)	1.33 (1.10,1.59)	1.44 (1.13,1.83)	ND

The midpoint generally corresponds to midpoints of World Health Organization BMI categories. Class III obese was divided into two sub‐classes (*a* and *b*) for the post‐term ≥42 week analysis given that data were available. Two studies 52, 72 were excluded from the nonlinear analyses as BMI was categorized in two groups only. Abbreviations: BMI, body mass index; CI, confidence interval; ND, no data available; OR, odds ratio.

### Meta‐analyses of post‐term birth ≥41 weeks of gestation

**Figure 4:**
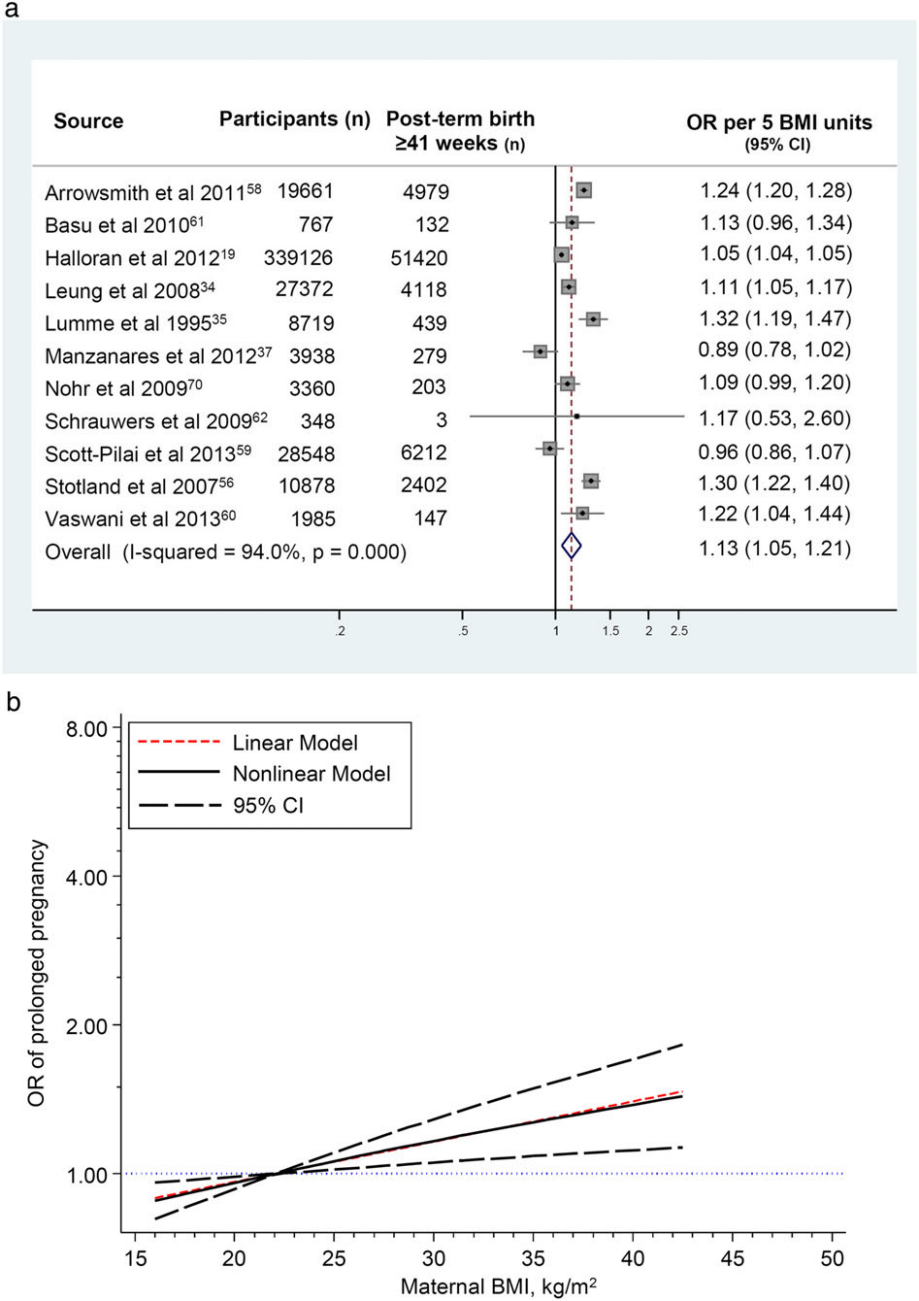
Linear and nonlinear dose–response association between maternal BMI and post‐term birth ≥41 weeks. (4a) Linear odds ratio per 5 maternal body mass index units. The squares and lines through the squares represent the study‐specific odds ratios and corresponding 95% confidence intervals. The dimension of the square is proportional to the weight of the study in the meta‐analysis. The diamond represents the summary odds ratio. (4b) Nonlinear and linear dose‐response analyses. analyses for post‐term birth. Linear model with data from all included studies; nonlinear model following sensitivity analysis and exclusion of Lumme *et al*. 35 (Fig. S5 and Table S9). [Colour figure can be viewed at wileyonlinelibrary.com]

The 11 studies with data for the meta‐analysis of births ≥41 weeks included 70,334 cases among 444,706 pregnancies (15.8% incidence). In the dose–response analysis, the OR for each 5 unit increase or decrease in BMI compared with the reference BMI midpoint was 1.13 (95% CI 1.05–1.21; heterogeneity *I* ^*2*^ = 94%, *p* < 0.001; Fig. 4a). Linearity of association between maternal BMI and birth ≥41 weeks is not rejected (*p* = 0.23, Table S6b). Assuming a linear association, this suggests a statistically significant decrease in odds of births ≥41 weeks for underweight BMI compared with the reference group and an increase for overweight and obese BMIs (Table 2 and Fig. 4b). This increasing linear association was also observed within the obesity classes, although to a lesser extent than for births ≥42 weeks (26%, 39% and 52% increased odds for classes I, II and III respectively; Table 2). There was no evidence of publication bias in the analyses of births ≥41 weeks (*p* = 0.16, Table S7).

### Sensitivity and heterogeneity analyses

Sensitivity analyses did not show any significant influence on linearity of any individual studies in the linear analyses for either post‐term categories (Tables S8 and S9) or in the nonlinear analysis for births ≥42 weeks (Table S8). For births ≥41 weeks, the sensitivity analyses for the nonlinear model detected that data from one study^35^ had an influence on linearity of the association between post‐term birth and maternal BMI (Table S9 and Fig. S5). The inclusion of data from all studies visually appeared to be nonlinear (Fig. S5); however, nonlinearity was not statistically significant (*p* = 0.065, Table S6c). When the data from this one study which was influencing linearity 35 were removed, the results showed a linear trend (Fig. 4b).

Meta‐regression exploring potential sources of heterogeneity identified that adjusting for the number of BMI exposure categories had the greatest influence on overall heterogeneity for births ≥42 weeks (*I* ^*2*^ reduced by 22.2%, from 98.1 to 75.95%, Table S10). Adjusting for additional variables in the meta‐regression did not have a substantial impact on overall heterogeneity for either post‐term outcomes. Sub‐group meta‐analyses for post‐term birth ≥42 weeks identified a significant reduction in heterogeneity (*I* ^*2*^ < 75%, *p* > 0.05, ≥3 studies) in the following categories: having three or four exposure categories, sample size between 1,000 and 10,000, controlling for induction of labour or caesarean delivery and controlling for hypertension or pre‐eclampsia (Table S10). The most relevant influence on heterogeneity in the sub‐group meta‐analyses of births ≥41 weeks was having four exposure categories (Table S11).

### Narrative summary of papers not included in the meta‐analysis

The 10 studies which had to be excluded from the meta‐analyses due to a lack of comparable data for pooling included two studies only reporting maternal weight and not BMI 36, 37; five did not report frequency data for participants and/or cases of post‐term birth 21, 38, 39, 40, 41, and three did not have comparable BMI reference groups (one combined all non‐obese 42, one combined underweight and recommended weight 43, and one combined recommended weight and overweight 44). Of the 10 studies not included in the meta‐analyses, six found a significantly increased risk of post‐term birth in obese women compared with the reference group 21, 37, 38, 39, 40, 44, while four did not find a significantly increased association 36, 41, 42, 43 (Table 3).

**Table 3:** Results of the studies included in the narrative summary

Study	Country of study	Sample size	Maternal weight exposure variable*	Post‐term variable	Results	Association with obesity	Primary reason not included in meta‐analysis
Abenhaim *et al*. 2007^41^	Canada	18,633	R: BMI 20 to 24.9; O1: BMI 30 to 39.9; O2: BMI >40	>42 weeks	O1: AOR 0.84 (95% CI 0.55–1.28); O2: AOR 0.76 (95% CI 0.19–3.10) Frequency data not provided	No significant difference	No frequency data provided
Al‐Rayyan *et al*. 2010^42^	Jordan	1,008	R: BMI <30; O: BMI >30	>42 weeks	R: *n* = 55, 10.6%; O: *n* = 54, 11.0%; Statistical analysis not reported	No difference	Non‐comparable BMI reference group
Briese *et al*. 2011^38^	Germany	243,571	R: BMI 18.5 to 24.9; O: BMI ≥30	Not defined	AOR 1.45 (95% CI 1.38–1.52) Frequency data not provided	Significantly increased	No frequency data provided
Caughey *et al*. 2009^21^	USA	119,162	R: ‘Not obese’; O: ‘Obese’ Not defined	≥41 weeks and ≥42 weeks	≥41 weeks AOR 1.26 (95% CI 1.16–1.37); ≥42 weeks AOR 1.20 (95% CI 0.99–1.46) Frequency data not provided	Significantly increased (41 weeks only)	No frequency data provided; non‐comparable BMI reference group
Denison *et al*. 2008^39^	Sweden	143,519	R: BMI 20 to <25; O1: BMI 30 to <35; O2: BMI ≥35	≥294 d (42 weeks)	Higher maternal BMI in 1st trimester increased post‐term (*p* < 0.001)	Significantly increased	No frequency data provided
Kistka *et al*. 2007^40^	USA	368,633	R: not defined; O: BMI >35	≥42 weeks	AOR 1.23 (95% CI 1.16–1.29) Frequency data not provided	Significantly increased	No frequency data provided
Mancuso *et al*. 1991^36^	Italy	160	R: BMI 15.2–26.6; O: BMI >30	>42 weeks	R: *n* = 1 O: *n* = 3 *p* > 0.05	No significant difference	Non‐comparable BMI reference group
Robinson *et al*. 2005^37^	Canada	142,404	R: 55 to 75 kg; O1: 90 to 120 kg; O2: >120 kg	>41 weeks	R: *n* = 4997, 6.3%; O1: *n* = 647, 6.9%; AOR 1.18 (95% CI 1.08–1.28); O2: *n* = 45, 5.8%; AOR 0.99 (95% CI 0.74–1.34)	Significantly increased (O1 only)	Maternal exposure weight
Sharief *et al*. 2000^36^	Iraq	40	R: ≤90 kg; O: >90 kg	Not defined	R: *n* = 3, 15%; O: *n* = 3, 15% Statistical analysis not reported	No difference	Maternal exposure weight
Usha Kiran *et al*. 2005^44^	Wales	8,350	R: BMI 20 to 30; O: BMI >30	>41 weeks	R: *n* = 2490, 32.5%; O: *n* = 278, 41.0%, OR 1.4 (95% CI 1.2–1.7)	Significantly increased	Non‐comparable BMI reference group

Abbreviations: AOR, adjusted odds ratio; BMI, body mass index; CI, confidence interval; O, obese weight group; OR, odds ratio; R, reference weight group.

## Discussion

This systematic review and meta‐analyses of over 4 million births have identified a significantly increasing association between maternal BMI and post‐term birth. This association increases in strength as BMI increases, with a substantial difference in effect size between obesity classifications: a difference of 33% in odds of post‐term birth ≥42 weeks and 26% for ≥41 weeks when comparing obesity classes I and III. This substantial increase in post‐term birth and associated risks for mothers in the highest obesity class presents a double burden of inequality. Women facing the greatest socio‐economic disadvantage 11 also have the highest level of pregnancy‐related risk, confirming that maternal obesity is both a clinical and public health priority for the wellbeing of women and their babies.

The mechanisms linking maternal BMI and post‐term birth are not fully understood. The onset of labour involves mechanical and hormonal interactions between the mother, foetus and placenta. The exact causal pathways remain unclear, and much of the evidence is based on animal models. This evidence suggests a number of potential mechanisms. Hormones are thought to play a key role in the pathway, including corticotrophin‐releasing hormone, oestrogen, progesterone, prostaglandins and oxytocin 45. Additionally, it is well established that women with obesity have increased inflammation, circulating leptin concentrations, insulin resistance, lipolysis and dyslipidaemia. These metabolic abnormalities have been hypothesized to influence the onset of spontaneous or oxytocin‐induced labour and uterine contractility 45. There is also evidence from one study in humans that shows that women with diabetes (including type 1 diabetes and gestational diabetes) had significantly reduced spontaneous myometrial contractility compared with women without diabetes, even after stimulation with oxytocin 46. Uterine biopsies identified reduced calcium channel expression and signalling among women with diabetes, and the authors concluded that this was likely to account for the reduced contractility in addition to a small but significant difference in myometrial mass 46. As obesity and diabetes are closely related, further exploration of myometrial contractility between women of different weight status' could provide further evidence for causal mechanisms of post‐term birth and obesity.

The heterogeneity in the relationship between degree of obesity and risk of post‐term birth is an important message for researchers, practitioners and policy makers. The implication of using one criterion to define the obese population is an attenuation of the true risk for the higher obesity classes. Despite the differences between obesity classes, pregnancy outcome data are often reported for one obese category. When pregnancy outcomes are reported by obesity class, a similar pattern is often reported. For example, the odds of pre‐term birth were reported to increase twofold from 1.6 (95% CI 1.4–1.8) for class I to 3.0 (95% CI, 2.3–3.9) for class III obesity 47. Similarly, the odds of GDM increased from 3.0 (95% CI 2.3–3.9) for class I to 5.6 (95% CI 4.3–7.2) for class III obesity 14. However, differentiating between obesity classes can be challenging. Although class III obesity is increasing at the most rapid rate over time 11, it only represents approximately 1% of pregnancies in the UK 11 and 4% in the USA 12. For population data to be powered for statistical significance, the sample size needs to be sufficient to detect enough cases in each obesity class. Our sub‐group meta‐analyses suggest that 100 cases of post‐term birth ≥42 weeks and 1000 cases for ≥41 weeks are required to detect significance, which may not always be feasible, even in national‐level datasets. When obesity classifications have to be combined for statistical power, there should be cautious interpretation of the results reflecting ‘obesity’ without consideration of the heterogeneous nature of obesity classifications. Additionally, the use of Asian‐specific rather than general population BMI criteria should be considered in future research. Although we did not identify any impact of using either definition on post‐term birth in this review, our analyses were limited as only one study had utilized the Asian‐specific criteria.

There are similar challenges with inconsistent use of post‐term birth categories. Meta‐analyses showed an increased association with maternal BMI and both post‐term categories and the highest odds for births ≥42 weeks. Although there is significantly increased risk for pregnancies progressing beyond 40 weeks 22, the greatest risk is in pregnancies with gestations ≥42 weeks 9, particularly for perinatal mortality and severe morbidities which require obstetric and neonatal intervention. Studies which combine post‐term birth categories are likely to underestimate the level of risk associated with maternal BMI.

A key strength of this review is overcoming the methodological challenges of investigating post‐term birth and maternal BMI. Analyses were performed throughout to explore the influence of methodological decisions, such as using unadjusted data and Asian‐specific BMI categories. The conversion of categorical BMI was necessary due to limited reporting of directly comparable obesity categories: 17 studies combined data for obesity classes I–III 19, 34, 35, 38, 42, 43, 44, 48, 49, 50, 51, 52, 53, 54, 55, 56, 57, three reported obesity classes I–III separately 58, 59, 60, four combined obesity classes I and II 20, 41, 61, 62, six combined classes II and III 39, 40, 63, 64, 65, 66, seven had further inconsistent non‐comparable categories such as combining overweight and obese 36, 37, 67, 68, 69, 70, 71, and two studies did not define their BMI categories 21, 72. The possible groups to combine for categorical analyses would have been further reduced when applying additional analysis criteria such as the gestational age stratification, definition of the reference BMI group, etc. Therefore, the conversion to continuous BMI allowed direct comparison of more studies overall than would have been possible by using a categorical meta‐analysis. To aid the interpretation of continuous BMI analyses, increments of 5 BMI units were used to allow back‐translation to approximate WHO categories. This allows for international comparison with other published research on maternal BMI and facilitates interpretation for clinical practice, public health and policy‐maker decisions which have a tendency to utilize BMI categories.

A further strength of this systematic review is the rigorous search strategy. It has been demonstrated that database searches alone are not sufficient for epidemiology systematic reviews 25, and the Meta‐analysis of Observational Studies in Epidemiology guidelines 73 recommend that additional searches may be necessary. We performed rigorous database searches including pilot and refinement of the search strategy by the research team, including an information scientist with expertise in database searching. This was supplemented by additional searches in our six‐stage search strategy to identify the full evidence base. Among the studies identified by using additional search methods, some were published in journals not indexed on the bibliographic databases and therefore would not have been identified by database searches alone. Furthermore, the post‐term data presented in a number of studies were not a primary outcome, rather one outcome among multiple adverse pregnancy outcomes being investigated. These studies did not include the post‐term search terms in the keywords, titles or abstracts and therefore would not have been identified by any search strategy using these terms. This rigorous search strategy was time consuming, although it resulted in an absence of publication bias. The method of searching (i.e. database, reference list or citation searches) was an *a priori* factor considered in the sub‐group meta‐analysis and meta‐regression to explore sources of heterogeneity between studies. While the method of searching did not impact on overall heterogeneity, the subgroup analyses suggest that the inclusion of studies identified through database searches was more likely to show statistically significant results in meta‐analysis than the studies identified by the additional searches (see Table S10 for example of analysis on post‐term ≥42 weeks). This result could have been due to more studies being included in the ≥42‐week sub‐group meta‐analysis identified by database searches (*n* = 12) compared with citation searches (*n* = 4) or reference list searches (*n* = 3). However, it could also suggest that database searches alone would result in positive publication bias by only identifying those studies more likely to show statistical significance. This result supports the Meta‐analysis of Observational Studies in Epidemiology guideline recommendation for supplementing database searches when carrying out systematic reviews of observational studies.

A limitation of systematic review methodology is reliance on the availability of published data which can impact on the analyses. The use of self‐reported last menstrual period or measured ultrasound scan is an important clinical factor influencing the assessment of gestational age, yet five studies did not specify methods of assessment for the ≥42 week meta‐analysis and a further seven for ≥41 weeks. Meta‐regression identified some factors considered to be important *a priori* which did not impact on the results, such as the use of self‐report or measured BMI. The use of self‐reported BMI among obese BMI groups is a frequent methodological criticism 74, yet had little influence in our meta‐regression analyses. Others have reported that the error caused by self‐report misclassification of BMI among overweight and obese women has minimal influence on the dose–response analyses for large for gestational age, gestational diabetes and preeclampsia 75. Therefore, the potential under‐reporting of self‐reported BMI appears to have little influence on large‐scale epidemiological analysis of maternal weight status and pregnancy outcomes. Additionally, 25 of the included studies did not report the ethnicity of their population and therefore, we could not explore this in the meta‐regression or sub‐group analysis which makes the generalizability across ethnicities challenging. However, one quarter of the studies were from the Middle East, Asia or South Africa which suggests that there was some ethnic diversity present in the populations rather than data originating from mainly White populations. Of the studies that reported ethnicity, eight studies described their population as mainly White, one as all Asian, one as mainly African, and four described a mix of ethnic groups in the population. The meta‐regression did explore country of study, and this did not impact on overall heterogeneity of results.

Maternal obesity is increasing internationally, and the daily challenges for clinical and public health practice will also continue to increase. The results of this systematic review and meta‐analyses add to the evidence‐base of increased risks associated with maternal obesity and can be used to inform preconception and pregnancy care. Policy makers should emphasize the importance of supporting women to reduce their BMI preconception and inter‐pregnancy to prevent the adverse outcomes associated with post‐term birth, such as perinatal and infant mortality. The increasing dose–response association also informs healthcare planning and commissioning of services, as the level and intensity of intervention required to prevent adverse outcomes associated with post‐term birth will differ according to BMI class. The data can also be used to inform the need for interventions such as induction of labour and caesarean delivery to prevent pregnancies progressing to post‐term. These procedures in obese populations also present clinical challenges and require increased planning, evidence‐based risk communication and shared decision‐making about birth plans. Any steps taken to support the health and wellbeing of women and their babies in relation to post‐term birth and associated risks should be informed by the dose–response association between the obesity classes. Further research which utilizes maternal BMI should also consider the heterogeneity within obesity populations and the need for adequately powered studies to explore pregnancy outcomes in the higher, less prevalent, obesity classes.

## Conclusions

Maternal obesity is having a significant impact on daily clinical practice. The association between maternal BMI and post‐term birth increases with increasing BMI, with the greatest odds among women in obesity class III and with post‐term birth ≥42 weeks. Pregnancies which progress beyond 42 weeks have significantly increased risk of adverse outcomes, including perinatal mortality. This presents a double burden of disease among women with morbid obesity, which is also associated with the highest levels of socio‐economic disadvantage compared with other BMI categories. Future maternal obesity research should consider the heterogeneity between obesity classes. Healthcare policy and practice should ensure that necessary interventions are in place to prevent the adverse outcomes associated with post‐term birth, considering the increased risk among the higher obesity classes.

## Conflict of interest statement

Dr Heslehurst has nothing to disclose.

Dr Vieira has nothing to disclose.

Dr Hayes has nothing to disclose.

Dr Crowe has nothing to disclose.

Mr Jones has nothing to disclose.

Ms Robalino has nothing to disclose.

Ms Slack has nothing to disclose.

Prof. Rankin has nothing to disclose.

## Supporting information


**Figure S1.** Translation of search terms across databases.
**Figure S2.** Adapted Newcastle‐Ottawa Scale.
**Figure S3.** Exploration of the use of adjusted or unadjusted data for post‐term birth (≥ 42 weeks and ≥41 weeks) meta‐analysis.
**Figure S4.** Sensitivity analysis for transforming Asian‐specific BMI reference criteria for the analysis of maternal BMI and post‐term birth (≥41 weeks gestation) using Asian‐specific BMI criteria for Leung et al. 2008.
**Figure S5.** Nonlinear dose‐response analysis for maternal BMI and post‐term birth ≥41 weeks, including all studies.
**Table S1.** Data extraction protocol.
**Table S2.** Screening: systematic review reference lists screened, and full papers screened and excluded.
**Table S3.** Details of included studies.
**Table S4.** Contacting authors for additional information.
**Table S5.** Quality scores for all included studies.
**Table S6.** Nonlinear meta‐analyses using cubic splines regression.
**Table S7.** Egger's test for publication bias for post‐term birth: ≥ 42 weeks and ≥41 weeks.
**Table S8.** Maternal BMI and post‐term birth ≥42 weeks sensitivity analysis.
**Table S9.** Maternal BMI and post‐term birth ≥41 weeks sensitivity analysis.
**Table S10.** Meta‐regression results for post‐term birth ≥42 weeks.
**Table S11.** Meta‐regression results for post‐term birth ≥41 weeks.

Supporting info item
