# Supplementary material for: Maternal body mass index and post‐term birth: a systematic review and meta‐analysis
Source: Obes Rev. 2017 Jan 13;18(3):293–308. doi: 10.1111/obr.12489 (PMC5324665; doi:10.1111/obr.12489)
Supplement: Supplementary file 1 — Supporting info item [file OBR-18-293-s001.docx]

**List of Supporting Information**

Figure S1: Translation of search terms across databases

Table S1: Data extraction protocol

Figure S2: Adapted Newcastle-Ottawa Scale

Table S2: Screening: systematic review reference lists screened, and full papers screened and excluded

Table S3: Details of included studies

Table S4: Contacting authors for additional information

Table S5: Quality scores for all included studies

Figure S3: Exploration of the use of adjusted or unadjusted data for post-term birth (≥ 42 weeks and ≥ 41 weeks) meta-analysis

Figure S4: Sensitivity analysis for transforming Asian-specific BMI reference criteria for the analysis of maternal BMI and post-term birth (≥41 weeks gestation) using Asian-specific BMI criteria for Leung et al 2008

Table S6: Nonlinear meta-analyses using cubic splines regression

Table S7: Egger’s test for publication bias for post-term birth: ≥ 42 weeks and ≥41 weeks

Table S8: Maternal BMI and post-term birth ≥ 42 weeks sensitivity analysis

Table S9: Maternal BMI and post-term birth ≥ 41 weeks sensitivity analysis

Figure S5: Nonlinear dose-response analysis for maternal BMI and post-term birth ≥41 weeks, including all studies

Table S10: Meta-regression results for post-term birth ≥ 42 weeks

Table S11: Meta-regression results for post-term birth ≥ 41 weeks

References: Reference list for the supporting information

**Figure S1: Translation of search terms across databases**

| **Embase (OVID) 1974 to 2015 Week 21**   1. *Pregnancy/ 2. Pregnan$. ti,ab. 3. Matern$. ti,ab. 4. Obes$. ti,ab. 5. (Body adj1 composition). ti,ab. 6. (BMI or Body mass index).ti,ab. 7. Weight.ti,ab. 8. (Post adj1 term$).mp. 9. Postterm$.mp. 10. (Post adj1 date$).mp. 11. Postdate$.mp. 12. (Prolonged adj1 pregnanc$).mp. 13. (Fetomaternal adj1 morbidity).mp. 14. Gestation.mp. 15. Postmaturity.mp. 16. (Post adj1 maturity).mp. 17. Cohort studies/ or longitudinal studies/ or follow-up studies/ or prospective studies/ or retrospective studies/ or cohort.ti,ab. or longitudinal.ti,ab. or prospective.ti,ab. or retrospective.ti,ab. 18. Case-Control Studies/ or Control Groups/ or Matched-Pair Analysis/ or ((case* adj5 control*) or (case adj3 comparison*) or control group*).ti,ab. 19. Cross-Sectional Studies/ or cross-sectional.ti,ab. or ("prevalence study" or "incidence study" or "prevalence studies" or "incidence studies" or "transversal studies" or "transversal study").ti,ab. 20. 1 or 2 or 3 21. 4 or 5 or 6 or 7 22. 8 or 9 or 10 or 11 or 12 or 13 or 14 or 15 or 16 23. 17 or 18 or 19 24. 20 and 21 and 22 and 23 25. Limit 24 to human 26. Limit 25 to female 27. *Body Mass Index/ 28. exp Overweight/ 29. *Obesity/ or *Obesity, Morbid/ 30. 21 or 27 or 28 or 29 31. *prolonged pregnancy/ 32. *pregnancy outcome/ 33. *pregnancy complication/ 34. 22 or 31 or 32 or 33 35. 20 and 23 and 30 and 34 36. 35 not 24 37. Limit 36 to human 38. Limit 37 to female   Note: .mp.=title, abstract, subject headings, heading word, drug trade name, original title, device manufacturer, drug manufacturer, device trade name, keyword  **PsycINFO (OVID) 1806 to May Week 3 2015**   1. *Pregnancy/ 2. Pregnan$. ti,ab. 3. Matern$. ti,ab. 4. Obes$. ti,ab. 5. (Body adj1 composition). ti,ab. 6. (BMI or Body mass index).ti,ab. 7. Weight.ti,ab. 8. (Post adj1 term$).mp. 9. Postterm$.mp. 10. (Post adj1 date$).mp. 11. Postdate$.mp. 12. (Prolonged adj1 pregnanc$).mp. 13. (Fetomaternal adj1 morbidity).mp. 14. Gestation.mp. 15. Postmaturity.mp. 16. (Post adj1 maturity).mp. 17. Cohort studies/ or longitudinal studies/ or follow-up studies/ or prospective studies/ or retrospective studies/ or cohort.ti,ab. or longitudinal.ti,ab. or prospective.ti,ab. or retrospective.ti,ab. 18. Case-Control Studies/ or Control Groups/ or Matched-Pair Analysis/ or ((case* adj5 control*) or (case adj3 comparison*) or control group*).ti,ab. 19. Cross-Sectional Studies/ or cross-sectional.ti,ab. or ("prevalence study" or "incidence study" or "prevalence studies" or "incidence studies" or "transversal studies" or "transversal study").ti,ab. 20. 1 or 2 or 3 21. 4 or 5 or 6 or 7 22. 8 or 9 or 10 or 11 or 12 or 13 or 14 or 15 or 16 23. 17 or 18 or 19 24. 20 and 21 and 22 and 23 25. Limit 24 to human 26. Limit 25 to female 27. *Body Mass Index/ 28. Exp Overweight/ 29. *Obesity/ or *Obesity, Morbid/ 30. 21 or 27 or 28 or 29 31. *obstetrical complications/ 32. *pregnancy outcomes/ 33. 22 or 31 or 32 34. 20 and 23 and 30 and 33 35. 34 not 24 36. Limit 36 to human 37. Limit 37 to female   Note:.mp.=title, abstract, heading word, table of contents, key concepts, original title, tests and measures  **CINAHL (EBSCO) 1981-May 2015**   1. MM pregnancy OR TI pregnan* OR TI matern* OR AB pregnan* OR AB matern* 2. MM body mass index OR MM obesity OR MH overweight+ OR TI obes* OR AB obes* OR TI body w1 composition OR AB body w1 composition OR TI bmi OR AB bmi OR TI body mass index OR AB body mass index 3. TI weight OR AB weight 4. 2 OR 3 5. TX ( post term* or postterm* ) OR TX ( (post w1 date*) or postdate* ) OR TX prolonged w1 pregnanc* OR TX fetomaternal w1 morbidity OR TX gestation OR TX postmaturity OR TX post w1 maturity OR MM “infant, postmature” OR MM “pregnancy outcomes” OR MM “pregnancy complications” 6. (MH “prospective studies”) OR (MH “case control studies+”) OR (MH “correlational studies”) OR (MH “nonconcurrent prospective studies”) OR (MH “cross sectional studies”) 7. TX (cohort w1 (study or studies)) 8. TX (observational w1 (study or studies)) 9. 6 OR 7 OR 8 10. 1 AND 4 AND 5 AND 9   **British Nursing Index (NHS HDAS) 1992-May 2015**   1. (pregnan* OR matern*).ti,ab. 2. PREGNANCY/ 3. 1 or 2 4. (obes* OR (body adj1 composition) OR bmi OR (body mass index) OR weight).ti,ab. 5. OBESITY/ 6. 4 OR 5 7. (postterm* OR (post adj1 term) OR postdate* OR (post adj1 date*) OR (prolonged adj1 pregnan*) OR (fetomaternal adj1 morbidity) OR gestation OR postmaturity OR (post adj maturity)).af 8. PREGNANCY : COMPLICATIONS/ 9. 7 OR 8 10. 3 AND 6 AND 9 |
| --- |

**Table S1: Data extraction protocol**

| **Reviewer** |  |
| --- | --- |
| **Title** |  |
| **Author and Year** |  |
| **Setting** | Location (region/city, country): Study name or dataset: |
| **Data collection time period** |  |
| **Endpoint and definition**  Author definition of gestation at delivery | Reference group: ……………… weeks gestation  Postdate birth 1: ……………….. weeks gestation  Postdate birth 2: ……………….. weeks gestation  Postdate birth 3: …………..…… weeks gestation |
| **Exposure definition** | BMI used to define groups? Yes / No Reference group defined as: …………..Kg/m^2^  BMI group(s) defined as:  Underweight: …………..……... Kg/m^2^  Overweight: …………………. Kg/m^2^  Obese group 1: ……………..… Kg/m^2^  Obese group 2: ……………….. Kg/m^2^  Obese group 3: ………………. Kg/m^2^  Obese group 4: ……….………. Kg/m^2^ |

**Methodology**:  Prospective Cohort  Retrospective Cohort **** Case Control

|  | Total group | Reference group | Under weight | Over weight | Obese group 1 | Obese group 2 | Obese group 3 | Obese group 4 |
| --- | --- | --- | --- | --- | --- | --- | --- | --- |
| Number Identified |  |  |  |  |  |  |  |  |
| Number Excluded |  |  |  |  |  |  |  |  |
| Final Number Included |  |  |  |  |  |  |  |  |
| All Subjects Accounted for? | Yes  No  Unclear | Yes  No  Unclear | Yes  No  Unclear | Yes  No  Unclear | Yes  No  Unclear | Yes  No  Unclear | Yes  No  Unclear | Yes  No  Unclear |

| Inclusion criteria (e.g. gestation at weight measurement, singleton etc) |  |
| --- | --- |
| Exclusion criteria |  |

| **Group Determination –** measure of maternal BMI |  Measured  Self Report  Unclear |
| --- | --- |
|  |  Medical Records  Prospectively collected for the study  Unclear |
| **Ascertainment of Outcome –** gestation at delivery |  Measured  Self Report  Unclear |
|  |  Medical Records  Prospectively collected for the study  Unclear |

**Baseline Characteristics reported?** Yes / No **(**if no do not complete, if yes populate with the data**)**

| **Characteristic**  (include definition and unit of measurement) | Total group | Reference group | Under weight | Over weight | Obese group 1 | Obese group 2 | Obese group 3 | Obese group 4 | P value |
| --- | --- | --- | --- | --- | --- | --- | --- | --- | --- |
| Maternal Age |  |  |  |  |  |  |  |  |  |
| Gestational Age at Booking |  |  |  |  |  |  |  |  |  |
| Ethnicity |  |  |  |  |  |  |  |  |  |
| Baseline BMI |  |  |  |  |  |  |  |  |  |
| Smoking Status |  |  |  |  |  |  |  |  |  |
| Illicit Drug Use |  |  |  |  |  |  |  |  |  |
| Alcohol Intake |  |  |  |  |  |  |  |  |  |
| Socio-Economic Status |  |  |  |  |  |  |  |  |  |
| Parity |  |  |  |  |  |  |  |  |  |
| Singleton Gestation |  |  |  |  |  |  |  |  |  |
| **Description of any differences between BMI groups:** | | | | | | | | | |

**Data Analysis:**

|  | **Number** | | | | **Crude**  **RR / OR**  (delete as applicable) | **…..% CI** | **P value** | **Adjusted**  **RR / OR**  (delete as applicable) | **…..% CI** | **P value** | **Factors adjusted for in analyses:** |
| --- | --- | --- | --- | --- | --- | --- | --- | --- | --- | --- | --- |
| **Outcome:**  **Postdate pregnancy**  **………wks** |  | **yes** | **no** | **Total** |  |  |  |  |  |  |  |
|  | Reference group |  |  |  |  |  |  |  |  |  |  |
|  | Under weight |  |  |  |  |  |  |  |  |  |  |
|  | Over weight |  |  |  |  |  |  |  |  |  |  |
|  | Obese |  |  |  |  |  |  |  |  |  | **Data Analysis methods:** |
|  | Obese group 2 |  |  |  |  |  |  |  |  |  |  |
|  | Obese group 3 |  |  |  |  |  |  |  |  |  |  |
|  | Obese group 4 |  |  |  |  |  |  |  |  |  |  |

**Figure S2: Adapted Newcastle-Ottatwa Scale ^1^ for Cohort Studies^#^**

| **Selection**  1) Representativeness of the exposed cohort (exposure in this context is maternal BMI risk group, e.g. obesity)  a) truly representative of the average pregnant population in the community *****  b) somewhat representative of the average pregnant population in the community *****  c) selected group of users (eg only first time pregnancy, only teenage pregnancy etc)  d) no description of the derivation of the cohort  2) Selection of the non exposed cohort (non-exposure is the maternal BMI group used as reference e.g. recommended BMI)  a) drawn from the same community as the exposed cohort*  b) drawn from a different source (E.g. different maternity unit, different specialist clinic, different time range for recruitment between BMI groups)  c) no description of the derivation of the non exposed cohort  3) Ascertainment of exposure (maternal weight status)  a) secure record ***** (Measured weight used)  b) validated self-report ***** (self-report with measured weight validation)  c) self report  d) no description  ~~4) Demonstration that outcome of interest was not present at start of study~~ ^##^  ~~a) yes~~ **~~*~~**  ~~b) no~~  **Comparability**  4) Comparability of cohorts on the basis of the design or analysis  a) study controls for induction of labour or caesarean ***** (either excluded or adjusted)  b) study controls for any additional factor *****  c) no factors controlled for  **Outcome**  5) Assessment of outcome (outcome is gestational age at delivery)  a) independent blind assessment/measured ***** (e.g. measurement (ultrasound scan) carried out prospectively for research purposes)  b) record linkage/measured ***** (e.g. retrospective routine hospital records of ultrasound scan to confirm gestational age)  c) self report (e.g. last menstrual period)  d) no description  6) Was follow-up long enough for outcomes to occur (until spontaneous onset of labour before post-term definition)  a) yes *****  b) no  7) Adequacy of follow up of cohorts  a) complete follow up - all subjects accounted for *****  b) subjects lost to follow up unlikely to introduce bias - small number lost to follow up >80 % (select an adequate %), or description provided of those lost *****  c) follow up rate < 80% and no description of those lost  d) no statement  **Total number of stars (out of a possible 8^###^):** |
| --- |

**Notes:**

**#** There were no true case control studies included in the systematic review where the case and control status was defined based on the case definition of the outcome variable (i.e. gestational age at delivery). Those studies which had been described by authors as case control, or that followed a case control method of selecting “cases” and “controls” (n=4) used the exposure status (BMI) to allocate case or control status. Therefore these studies used the pre/early pregnancy baseline exposure status to define the groups and followed the women for the duration of their pregnancy until birth to ascertain delivery outcomes (either prospectively or retrospectively). These study designs better fit the cohort design Quality Assessment Scale and therefore this has been used for all included studies.

## Question 4 “Demonstration that outcome of interest was not present at start of study” is not applicable to gestational age at delivery outcomes as women are identified in early pregnancy using their pre/early pregnancy BMI and their pregnancy outcomes are not known at the start of the study. Therefore this item has been removed from the scale

### The denominator value for the maximum number of stars a study can be awarded has been reduced from 9 to 8 due to the removal of the original question 4.

**Table S2: Screening: systematic review reference lists screened, and full papers screened and excluded**

**Table S2a: Reference lists of systematic reviews screened**

| **Review** | **Number of references screened** |
| --- | --- |
| Bogaerts et al 2013 ^2^ | 70 |
| Castro and Avina 2002 ^3^ | 31 |
| Catalano and Ehrenberg 2006 ^4^ | 55 |
| Caughey et al 2008 ^5^ | 99 |
| Gülmezoglu et al 2012 ^6^ | 137 |
| Heslehurst et al 2008 ^7^ | 85 |
| Linne 2004 ^8^ | 93 |
| Lutsiv et al 2015 ^9^ | 94 |
| McDonald et al 2010 ^10^ | 102 |
| Nuthulapaty and Rouse 2004 ^11^ | 97 |
| Torloni et al 2009 ^12^ | 77 |
| Vasudevan et al 2011 ^13^ | 50 |
| Walker and Gan 2015 ^14^ | 8 |
| Wolfe 1998 ^15^ | 10 |

**Table S2b: Full details of studies excluded following full paper review**

| **References of studies screened in full and excluded** | |
| --- | --- |
| **Exclusion Reason** | **Reference Number** |
| Abstract/poster only | 1-18 |
| *Abstract authors contacted*  *Unable to contact* | *1-5, 8-18*  *6-7* |
| BMI at term/late pregnancy | 19-22 |
| BMI not an exposure | 23-32 |
| Multiple gestations only | 33 |
| Not post term | 34-142 |
| Not primary research | 143-159 |
| Restricted subsample of maternity population | 160-169 |
| Unpublished student dissertation | 170 |
| **Reference list of excluded studies** | |
| 1. Cidade, D. G., P. R. Margotto, A. C. B. S. Guedes, A. A. Rocha, F. R. Assis, F. F. Cardoso, R. C. R. Lemes, V. T. M. Borges and J. C. Peracoli (2012). "High prevalence of pre-pregnancy overweight and obesity associated with maternal and perinatal complications." Pregnancy Hypertension 2 (3): 323. 2. Darsareh, F. and S. Nourbakhsh (2012). "Pre-pregnancy body mass index and the risk of prolonged pregnancy." International Journal of Gynecology and Obstetrics 119: S756. 3. Guariglia, L., P. Ciliberti, S. Buongiorno, A. Alessio, E. Nobili, M. Tintoni, P. Rosati and G. Capelli (2013). "Risk factors in prolonged pregnancy." Journal of Perinatal Medicine 41. 4. Hallaron, D. R., N. Marshall, Y. W. Cheng and A. B. Caughey (2012). "Effect of obesity on induction across gestational age." American Journal of Obstetrics and Gynecology 1): S250. 5. Idris, N. and K. N. C. Nyan (2012). "The association of maternal obesity and gestational weight gain with obstetric and neonatal outcomes among parturients in Seremban, Malaysia." BJOG: An International Journal of Obstetrics and Gynaecology 119: 75. 6. Kapoor, D. and S. Rajendran (2013). "Can we improve care and outcomes of pregnancy in women with morbid obesity?" BJOG: An International Journal of Obstetrics and Gynaecology 120: 30-31. 7. Kapoor, D., J. Davison and S. Rajendran (2013). "Audit on care and outcome of pregnancy in women with morbid obesity." Archives of Disease in Childhood: Fetal and Neonatal Edition 98. 8. Lam, S., L. Kindinger and L. Phelan (2012). "Weight gain in pregnancy." Archives of Disease in Childhood: Fetal and Neonatal Edition 97: A114. 9. Marshall, N. E., C. Guild, Y. W. Cheng, A. B. Caughey and D. R. Halloran (2012). "Impact of maternal BMI on induction of labor." American Journal of Obstetrics and Gynecology 1): S147. 10. Marshall, N. E., J. M. Snowden, P. F. O'Tierney-Ginn, K. Melsap, J. Chung, E. Main, W. Gilbert and A. B. Caughey (2013). "Influence of fetal sex, maternal obesity, and gestational weight gain on perinatal outcomes." Reproductive Sciences 1): 309A-310A. 11. Martin, K., R. M. Grivell, L. N. Yelland and J. M. Dodd (2013). "Gestational diabetes mellitus among women who are overweight and obese: The effect of BMI category." Obesity Research and Clinical Practice 7: 11. 12. Nohr, E. A. (2012). "Obesity in pregnancy. Outcomes in the mother and child." Obesity Facts 5: 20. 13. O'Dwyer, V., J. Hogan, N. Farah, M. M. Kennelly, B. Stuart and M. J. Turner (2012). "Changes in maternal body composition during pregnancy." Archives of Disease in Childhood: Fetal and Neonatal Edition 97: A47. 14. Oniya, O., K. Hanretty, J. Gibson and K. Guerrero (2010). "Audit of pregnancy outcomes and co-morbidities in the obese population." Obesity Reviews 11 (11): 830. 15. Redfearn, C. F., S. Wandiembe and E. Oteng-Ntim (2012). "Quantification of healthcare costs of obesity in pregnancy: A retrospective observational cohort study." Archives of Disease in Childhood: Fetal and Neonatal Edition 97: A110. 16. Schuster, M. Neubert, A. Kirchner, L Paglia, M. The impact of body mass index on pregnancy complications. 2015. American Journal of Obstetrics and Gynaecology. 1): S421. 17. Suresh, A., A. Liu, A. Poulton, A. Quinton, Z. Amer, M. Mongelli, A. Martin, R. Benzie, M. Peek and R. Nanan (2012). "Comparison of maternal abdominal subcutaneous fat thickness and body mass index as markers for pregnancy outcomes: A stratified cohort study." Australian and New Zealand Journal of Obstetrics and Gynaecology 52(5): 420-426. 18. Trombe, K. S. D., H. Bettiol, R. C. Cavalli, M. R. P. Gutierrez, M. A. Barbieri, C. Grandi and V. C. Cardoso (2014). "Association between maternal pre-pregnancy body mass index and size at birth in Ribeirao Preto, Sao Paulo, Brazil." Archives of Disease in Childhood 99: A240. 19. Fatima, S. A. Rehman, S. A. Gangat, A. Kamal, Z. Ahmad (2011). “To compare maternal and fetal outcome in obese verses non-obese labouring mothers.” Journal University Medical and Dental College 2(2): Jul-Dec 2011. 20. Mamula, O., N. S. Severinski, M. Mamula and S. Severinski (2009). "Complications during pregnancy, labor and puerperium in women with increased BMI at pregnancy term." Central European Journal of Medicine 4(1): 71-75. 21. Metzger, B. E. (2010). "Hyperglycaemia and adverse pregnancy outcome (HAPO) study: Associations with maternal body mass index." BJOG: An International Journal of Obstetrics and Gynaecology 117(5): 575-584. 22. Pongthai, S. (1990). "Labour and delivery of obese parturients." Journal of the Medical Association of Thailand 73 Suppl 1: 52-56. 23. Campbell MK, Ostbye T, Irgens LM. Postterm birth: risk factors and outcomes in a 10-year cohort of Norwegian births. Obstet Gynecol1997;89:543-8. 24. Caughey and Bishop, Maternal complications of pregnancy increase beyond 40 weeks of gestation in low-risk women. J Perinatol 2006:26:540-545 25. Caughey et al Maternal and obstetric complications of pregnancy are associated with increasing gestational age at term. Am j obstet gynecol 2007 196(2)155 e1-6 26. Collins, J. W., N. F. Schulte, L. George and A. Drolet (2000). "Postterm delivery among African Americans, Mexican Americans and Whites in Chicago." Ethnicity & disease 11(2): 181-187. 27. Fleten, C., H. Stigum, P. Magnus and W. Nystad (2010). "Exercise during pregnancy, maternal prepregnancy body mass index, and birth weight." Obstetrics & Gynecology 115(2, Part 1): 331-337. 28. Greve et al Maternal and perinatal complications by day of gestation after spontaneous labor at 40-42 weeks of gestation. Acta obstetricia Gynecol Scand 2011 Aug; 90: 852-6 29. Jukic et al. (2013) “Length of Human Pregnancy and contributors to its natural variation” Human Reproduction 2013 Oct;28(10):2848-2855. 30. Olesen, A. W., J. G. Westergaard and J. Olsen (2003). "Perinatal and maternal complications related to postterm delivery: a national register-based study, 1978-1993." American journal of obstetrics and gynecology 189(1): 222-227. 31. Shea, K. M., A. J. Wilcox and R. E. Little (1998). "Postterm delivery: a challenge for epidemiologic research." Epidemiology 9(2): 199-204. 32. Zareen, N., S. Naqvi, N. Majid and H. Fatima (2009). "Perinatal outcome in high risk pregnancies." Journal of the College of Physicians and Surgeons Pakistan 19(7): 432-435. 33. Moore, E. and J. Sumners (2012). "Impact of maternal pre-pregnancy body mass index on pregnancy and infant outcomes in triplet gestations." American Journal of Obstetrics and Gynecology 1): S67. 34. Abu Yaacob S, Saad FA, Sharara HA, Khalifa L, Manther AA, Rashed YA. The effect of obesity in pregnancy on perinatal outcome in Qatar. Qatar Medical Journal 2002;11:32-5 35. Alanis, M.C., Goodnight, W.H., Hill, E.G., Robinson, C.J., Villers, M.S. and Johnson, D.D. (2010) Maternal super-obesity (body mass index > or =50) and adverse pregnancy outcomes. Acta Obstetricia et Gynecologica Scandinavica, 89, 924-930. 36. Andreasen, K. R., M. L. Andersen and A. L. Schantz (2004). "Obesity and pregnancy." Acta obstetricia et gynecologica Scandinavica 83(11): 1022-1029. 37. Athukorala, C., Rumbold, A.R., Willson, K.J. and Crow- ther, C.A. (2010) The risk of adverse pregnancy out- comes in women who are overweight or obese. BMC Pregnancy Childbirth, 10, 56. 38. Baeten, J. M., E. A. Bukusi and M. Lambe (2001). "Pregnancy complications and outcomes among overweight and obese nulliparous women." American Journal of Public Health 91(3): 436. 39. Barau G, Robillard PY, Hulsey TC, Dedecker F, Laffite A, Gérardin P, et al. Linear association between maternal pre-pregnancy body mass index and risk of caesarean section in term deliveries. Br J Obstet Gynaecol 2006;113:1173-7. 40. Baron CM, Girling LG, Mathieson AL, Menticoglou SM, Seshia MM, Cheang MS, Mutch WA: Obstetrical and neonatal outcomes in obese parturients. J Matern Fetal Neonatal Med 2010, 23(8):906–913. 41. Beyer et al., 2011. Obesity decreases the chance to deliver spontaneously Archives of Gynecology and Obstetrics, 283 (2011), pp. 981–988 42. Bianco, A. T., S. W. Smilen, Y. Davis, S. Lopez, R. Lapinski and C. J. Lockwood (1998). "Pregnancy outcome and weight gain recommendations for the morbidly obese woman." Obstetrics & Gynecology 91(1): 97-102. 43. Black, M., et al., Maternal overweight and obesity account for a greater proportion of adverse pregnancy outcomes than does gestational diabetes (GDM) defined by International Association of Diabetes in Pregnancy Study Groups (IADPSG) criteria. American Journal of Obstetrics and Gynecology, 2012. 1): p. S121-S122 44. Bowers, D. and W. R. Cohen (1999). "Obesity and related pregnancy complications in an inner-city clinic." Journal of Perinatology 19(3): 216-219. 45. Burstein E, Levy A, Mazor M, Wiznitzer A, Sheiner E. Pregnancy outcome among obese women: a prospective study. Am J Perinatol 2008;25(9):561–6. 46. Callaway LK, Prins JB, Chang AM, McIntyre HD. The prevalence and impact of overweight and obesity in an Australian obstetric population. Med J Aust 2006;184(2):56–9. 47. Choi et al 2011 The effects of pre-pregnancy body mass index and gestational weight gain on perinatal outcomes in Korean women: a retrospective cohort study. Reproductive Biology and Endocrinology 2011, 9:6 48. Choudhry H, Choudhry A, Azam N, Jan S. Effects of obesity on pregnancy and its outcome. Pak Armed Forces J 2009;59:315-9. 49. Clausen T, Oyen N, Henriksen T. Pregnancy complications by overweight and residential area. A prospective study of an urban Norwegian cohort. Acta Obstet Gynecol Scand2006;85:526-33 50. Cnattingius, S., R. Bergström, L. Lipworth and M. S. Kramer (1998). "Prepregnancy weight and the risk of adverse pregnancy outcomes." New England Journal of Medicine 338(3): 147-152. 51. Crane SS, Wojtowycz MA, Dye TD, Aubry RH, Artal R. Association between prepregnancy obesity and the risk of cesarean delivery. Obstet Gynecol 1997;89:213-6. 52. Dodd JM, Grivell RM, Nguyen A, Chan A, Robinson JS. Maternal and perinatal health outcomes by body mass index category. Aust N Z J Obstet Gynaecol 2011;51:136–40. 53. Doherty DA, Magann EF, Francis J, Morrison JC, Newnham JP. Pre-pregnancy body mass index and pregnancy outcomes. Int J Gynaecol Obstet 2006;95:242–247. 54. Driul L, Cacciaguerra G, Citossi A, Martina MD, Peressini L, Marchesoni D. Prepregnancy body mass index and adverse pregnancy outcomes. Arch Gynecol Obstet2008;278:23-6 55. Edwards, L. E., W. L. Hellerstedt, I. R. Alton, M. Story and J. H. Himes (1996). "Pregnancy complications and birth outcomes in obese and normal-weight women: effects of gestational weight change." Obstetrics & Gynecology 87(3): 389-394. 56. Ekblad U, Grenman S. Maternal weight, weight gain during pregnnacy and prenancy outcome Int J Gynaecol Obstet 1992;39:277-283 57. El-Chaar et al. The Impact of Increasing Obesity Class on Obstetrical Outcomes MARCH JOGC 2013. p224 58. El-Chaar, D., S. A. Finkelstein, X. Tu, D. B. Fell, L. Gaudet, J. Sylvain, G. Tawagi, S. W. Wen and M. Walker (2013). "The impact of increasing obesity class on obstetrical outcomes." Journal of obstetrics and gynaecology Canada : JOGC = Journal d'obstetrique et gynecologie du Canada : JOGC 35(3): 224-233. 59. Flick, A.A., et al., Excessive weight gain among obese women and pregnancy outcomes. American Journal of Perinatology, 2010. 27(4): p. 333-8. 60. Green, C. and D. Shaker (2011). "Impact of morbid obesity on the mode of delivery and obstetric outcome in nulliparous singleton pregnancy and the implications for rural maternity services." Australian & New Zealand Journal of Obstetrics & Gynaecology 51(2): 172-174. 61. Halloran, D.R., et al., Effect of revised IOM weight gain guidelines on perinatal outcomes. Journal of Maternal-Fetal & Neonatal Medicine, 2011. 24(3): p. 397-401 62. Hancke et al Pre-pregnancy obesity compromises obstetric and neonatal outcomes. Journal of Perinatal Medicine. 2014. Volume 43, Issue 2, Pages 141–146 63. Hauger, M. S., Gibbons, L., Vik, T. and Belizan, J. M. (2008), Prepregnancy weight status and the risk of adverse pregnancy outcome. Acta Obstetricia et Gynecologica Scandinavica, 87: 953–959. 64. Hoffman, C. S., L. C. Messer, P. Mendola, D. A. Savitz, A. H. Herring and K. E. Hartmann (2008). "Comparison of gestational age at birth based on last menstrual period and ultrasound during the first trimester." Paediatric and Perinatal Epidemiology 22(6): 587-596. 65. Iqbal, Nargis, et al. "A Study of Association of Obesity with Maternal Complications." Annals of King Edward Medical University 19.3 (2014). 66. J.A Kusin, S Kardjati, U.H Renvqist. Maternal body mass index: the functional significance during reproduction. 1994. European Journal of Clinical Nutrition 48 Suppl 3: S56-67. 67. Jensen DM, Damm P, SA˜¸rensen B, MA˜¸lsted-Pedersen L, Westergaard JG, et al. (2003) Pregnancy outcome and prepregnancy body mass index in 2459 glucose tolerant Danish women. American Journal of Obstetrics and Gynecology 189:239–244. 68. Kabiru, W. and B. D. Raynor (2004). "Obstetric outcomes associated with increase in BMI category during pregnancy." American journal of obstetrics and gynecology 191(3): 928-932. 69. Kalk P, Guthmann F, Krause K, Relle K, Godes M, Gossing G, Halle H, Wauer R, Hocher B. Impact of maternal body mass index on neonatal outcome. Eur J Med Res 2009;14:216–222. 70. Kaplan-Sturk etal. Outcome of deliveries in healthy but obese women: obesity and delivery outcome BMC Research Notes 2013, 6:50 71. Kerrigan AM, Kingdon C. Maternal obesity and pregnancy: aretrospective study. Midwifery 2010;26:138–46. 72. Ketterlinus, R. D., S. H. Henderson and M. E. Lamb (1990). "Maternal age, sociodemographics, prenatal health and behavior: Influences on neonatal risk status." Journal of Adolescent Health Care 11(5): 423-431. 73. Kitsantas, P. and L.R. Pawloski, Maternal obesity, health status during pregnancy, and breastfeeding initiation and duration. Journal of Maternal-Fetal & Neonatal Medicine, 2010. 23(2): p. 135-41. 74. Kominiarek MA, Vanveldhuisen P, Hibbard J, Landy H, Haberman S, Learman, L, Wilkins I, Bailit J, Branch W, Burkman R, Gonzales-Quintero VH, Gregory K, Hatjis C, Hoffman M, Ramirez M, Reddy UM, Troendle J, Zhang J for the Consortium on Safe Labor: The maternal body mass index: a strong association with delivery route. Am J Obstet Gynecol 2010, 203(3):264. e261–264.e267. 75. Kominiarek MA, Zhang J, Vanveldhuisen P, Troendle J, Beaver J, Hibbard JU: Contemporary labor patterns: the impact of maternal body mass index. Am J Obstet Gynecol 2011, 205(3):244.e241–244.e248. 76. Korapin Rudtanasudjatum, M. D., and Teerapat Chullapram. "The Association between Obesity and the Risk of Cesarean Delivery and other Adverse Pregnancy Outcomes in Singleton Term Pregnancies." 77. Kumar, A., K. Chaudhary and S. Prasad (2010). "Maternal indicators and obstetric outcome in the north Indian population: A hospital-based study." Journal of Postgraduate Medicine 56(3): 192-195. 78. Kumari, A. S. (2001). "Pregnancy outcome in women with morbid obesity." International Journal of Gynecology & Obstetrics 73(2): 101-107. 79. Le Thai, N., G. Lefebvre, et al. (1992). "[Pregnancy and obesity. A case control study of 140 cases]." Journal de Gynecologie, Obstetrique et Biologie de la Reproduction 21(5): 563-567. 80. Liu, X., Du, J., Wang, G., Chen, Z., Wang, W. and Xi, Q. (2011) Effect of pre-pregnancy body mass index on ad- verse pregnancy outcome in north of China. Archives of Gynecology and Obstetrics, 283, 65-70. 81. Lu, G. C., D. J. Rouse, M. DuBard, S. Cliver, D. Kimberlin and J. C. Hauth (2001). "The effect of the increasing prevalence of maternal obesity on perinatal morbidity." American journal of obstetrics and gynecology 185(4): 845-849. 82. M.A. Kominiarek, J. Zhang, P. Vanveldhuisen, J. Troendle, J. Beaver, J.U. Hibbard. Contemporary labor patterns: the impact of maternal body mass index. American Journal of Obstetrics and Gynecology, 205 (2011), pp. 244–248 83. M.A. Kominiarek, P. Vanveldhuisen, J. Hibbard, et al. The maternal body mass index: a strong association with delivery route American Journal of Obstetrics and Gynecology, 203 (2010), pp. 264–267 84. Magann et al. Pregnancy, obesity, gestational weight gain, and parity as predictors of peripartum complications. Archives of Gynecology and Obstetrics 2010 284:1754 85. Marshall, N. E., C. Guild, Y. W. Cheng, A. B. Caughey and D. R. Halloran (2014). "Racial disparities in pregnancy outcomes in obese women." Journal of Maternal-Fetal and Neonatal Medicine 27(2): 122-126. 86. Marshall, N. E., C. Guild, Y. W. Cheng, A. B. Caughey and D. R. Halloran (2014). "The effect of maternal body mass index on perinatal outcomes in women with diabetes." American Journal of Perinatology 31(3): 249-256. 87. Mobasheri, E. and M. J. Golalipour (2007). "The effect of pre-pregnancy body mass index on gestational weight gain and pregnancy outcome in Gorgan, North Iran." Journal of Medical Sciences 7(5): 905-908. 88. Mochhoury, L., R. Razine, J. Kasouati, M. Kabiri and A. Barkat (2013). "Body mass index, gestational weight gain, and obstetric complications in Moroccan population." Journal of pregnancy 2013: 379461. 89. Munim, S. and H. Maheen (2012). "Association of gestational weight gain and pre-pregnancy body mass index with adverse pregnancy outcome." Journal of the College of Physicians and Surgeons--Pakistan : JCPSP 22(11): 694-698. 90. Naeye, R. L. (1990). "Maternal body weight and pregnancy outcome." American Journal of Clinical Nutrition 52(2): 273-279. 91. Nanyonjo, R. D., S. B. Montgomery, N. Modeste and E. Fujimoto (2008). "A secondary analysis of race/ethnicity and other maternal factors affecting adverse birth outcomes in San Bernardino County." Maternal and Child Health Journal 12(4): 435-441. 92. Narchi, H. and Skinner, A. (2010) Overweight and ob- esity in pregnancy do not adversely affect neonatal out- comes: New evidence. Journal of Obstetrics and Gynae- cology, 30, 679-686. 93. Nohr, E. A., M. Vaeth, J. L. Baker, T. I. A. Sorensen, J. Olsen and K. M. Rasmussen (2008). "Combined associations of prepregnancy body mass index and gestational weight gain with the outcome of pregnancy." American Journal of Clinical Nutrition 87(6): 1750-1759. 94. Nohr, E. A., M. Vaeth, J. L. Baker, T. I. A. Sorensen, J. Olsen and K. M. Rasmussen (2009). "Pregnancy outcomes related to gestational weight gain in women defined by their body mass index, parity, height, and smoking status." American Journal of Clinical Nutrition 90(5): 1288-1294. 95. Ogunyemi D, Hullett S, Leeper J, Risk A. Prepregnancy body mass index, weight gain during pregnancy, and perinatal outcome in a rural black population. J Matern Fetal Med1998;7:190-3 96. Oken, E., K. P. Kleinman, S. F. Olsen, J. W. Rich-Edwards and M. W. Gillman (2004). "Associations of seafood and elongated n-3 fatty acid intake with fetal growth and length of gestation: Results from a US pregnancy cohort." American Journal of Epidemiology 160(8): 774-783. 97. Olsen, S. F., M. L. Osterdal, J. D. Salvig, U. Kesmodel, T. B. Henriksen, M. Hedegaard and N. J. Secher (2006). "Duration of pregnancy in relation to seafood intake during early and mid pregnancy: Prospective cohort." European Journal of Epidemiology 21(10): 749-758. 98. Ovesen, P., Rasmussen, S. and Kesmodel, U. (2011) Ef- fect of prepregnancy maternal overweight and obesity on pregnancy outcome. Obstetrics & Gynecology, 118, 305- 312. 99. Park, J. H., B. E. Lee, H. S. Park, E. H. Ha, S. W. Lee and Y. J. Kim (2011). "Association between pre-pregnancy body mass index and socioeconomic status and impact on pregnancy outcomes in Korea." Journal of Obstetrics and Gynaecology Research 37(2): 138-145. 100. Patel, R. R., P. Steer, P. Doyle, M. P. Little and P. Elliott (2004). "Does gestation vary by ethnic group? A London-based study of over 122 000 pregnancies with spontaneous onset of labour." International Journal of Epidemiology 33(1): 107-113. 101. Perlow JH, Morgan MA, Montogomery D, Towers CV, Porto M. Perinatal outcome in pregnancy complicated by massive obesity. Am J Obstet Gynecol 1992;167:958–962. 102. Raja, U. A., T. McAree, P. Bassett and S. Sharma (2012). "The implications of a raised maternal BMI: A DGH experience." Journal of Obstetrics and Gynaecology 32(3): 247-251. 103. Ramos, G. A. and A. B. Caughey (2005). "The interrelationship between ethnicity and obesity on obstetric outcomes." American journal of obstetrics and gynecology 193(3): 1089-1093. 104. Rantakallio et al Maternal build and pregnancy outcome. Journal of Clinical Epidemiology, Volume 48, Issue 2, February 1995, Pages 199–207 105. Rayco-Solon, P., A. J. Fulford and A. M. Prentice (2005). "Maternal preconceptional weight and gestational length." American Journal of Obstetrics & Gynecology 192(4): 1133-1136. 106. Rayis, D. A. Abbaker, A. O. Salih, Y. Adam, I. Obesity and pregnancy outcome in Khartoum, Sudan. 2011. International Journal of Gynaecology and Obstetrics 113(2): 160-161. 107. Reddy, U. M., S. K. Laughon, L. Sun, J. Troendle, M. Willinger and J. Zhang (2010). "Prepregnancy risk factors for antepartum stillbirth in the United States." Obstetrics and Gynecology 116(5): 1119-1126. 108. Reiss, Katharina, et al. "Contribution of overweight and obesity to adverse pregnancy outcomes among immigrant and non-immigrant women in Berlin, Germany." The European Journal of Public Health (2015): ckv072. 109. Retnakaran, R., C. Ye, A. J. G. Hanley, P. W. Connelly, M. Sermer, B. Zinman and J. K. Hamilton (2012). "Effect of maternal weight, adipokines, glucose intolerance and lipids on infant birth weight among women without gestational diabetes mellitus." Cmaj 184(12): 1353-1360. 110. Rezaie, Masomeh, Roonak Shahoei, and Shoaleh Shahghebi. "The effect of maternal body mass index on the delivery route in nulliparous women." Journal of Public Health 5.12 (2013): 493-497. 111. Ricart, W., J. Lopez, J. Mozas, A. Pericot, M. A. Sancho, N. Gonzalez, M. Balsells, R. Luna, A. Cortazar, P. Navarro, O. Ramirez, B. Flandez, L. F. Pallardo, A. Hernandez-Mijas, J. Ampudia, J. M. Fernandez-Real and R. Corcoy (2005). "Body mass index has a greater impact on pregnancy outcomes than gestational hyperglycaemia." Diabetologia 48(9): 1736-1742. 112. Roman, H., P. Y. Robillard, T. C. Hulsey, A. Laffitte, K. Kouteich, L. Marpeau and G. Barau (2007). "Obstetrical and neonatal outcomes in obese women." West Indian Medical Journal 56(5): 421-426. 113. Rosenberg TJ, Garbers S, Chavkin W, Chiasson MA. Prepregnancy weight and adverse perinatal outcomes in an ethnically diverse population. Obstet Gynecol. 2003;102:1022–7. 114. Rozlan et al The Association of Gestational Weight Gain and the Effect on Pregnancy Outcome Defined by BMI Group among Women Delivered in Hospital Kuala Lumpur (HKL), Malaysia: A Retrospective Study. Asian Journal of Clinical Nutrition4.4 (2012): 160-167 115. Saereeporncharenkul, K. (2011) Correlation of BMI to pregnancy outcomes in Thai women delivered in Rajavithi Hospital. Journal of the Medical Association of Thailand, 94, S52-S58. 116. Sahu MT, Agarwal A, Das V, Pandey A. Impact of maternal body mass index on obstetric outcome. J Obstet Gynaecol Res 2007;33:655-9. 117. Sarkar, R. K., S. M. Cooley, J. C. Donnelly, T. Walsh, C. Collins and M. P. Geary (2007). "The incidence and impact of increased body mass index on maternal and fetal morbidity in the low-risk primigravid population." Journal of Maternal-Fetal and Neonatal Medicine 20(12): 879-883. 118. Schummers, Laura, et al. "Risk of adverse pregnancy outcomes by prepregnancy body mass index: a population-based study to inform prepregnancy weight loss counseling." Obstetrics & Gynecology 125.1 (2015): 133-143. 119. Sebire, N. J., M. Jolly, J. Harris, L. Regan and S. Robinson (2001). "Is maternal underweight really a risk factor for adverse pregnancy outcome? A population-based study in London." British Journal of Obstetrics and Gynaecology 108(1): 61-66. 120. Sebire, N. J., M. Jolly, J. P. Harris, J. Wadsworth, M. Joffe, R. W. Beard, L. Regan and S. Robinson (2001). "Maternal obesity and pregnancy outcome: A study of 287 213 pregnancies in London." International Journal of Obesity 25(8): 1175-1182. 121. Shah, D., A. Tay, A. Desai, M. Parikh, M. Nauta and W. Yoong (2011). "The obstetric performance of Chinese immigrants residing in the UK." Journal of Obstetrics and Gynaecology 31(6): 480-482. 122. Sherrard A, Platt RW, Vallerand D, Usher RH, Zhang X, Kramer MS. Maternal anthropometric risk factors for caesarean delivery before of after onset of labour. Br J Obstet Gyneacol 2007;114:1088-96. 123. Stamnes Koepp, U. M., L. Frost Andersen, K. Dahl-Joergensen, H. Stigum, O. Nass and W. Nystad (2012). "Maternal pre-pregnant body mass index, maternal weight change and offspring birthweight." Acta Obstetricia et Gynecologica Scandinavica 91(2): 243-249. 124. Suresh, A., A. Liu, A. Poulton, A. Quinton, Z. Amer, M. Mongelli, A. Martin, R. Benzie, M. J. Peek and R. Nanan (2013). "Is maternal abdominal subcutaneous fat thickness a better marker for pregnancy outcomes than body mass index: A stratified cohort study." BJOG: An International Journal of Obstetrics and Gynaecology 120: 94-95. 125. Syngelaki A, Bredaki F.E, Vaikousi E, Maiz N.a, Nicolaides K.H (2011) Body Mass Index at 11–13 Weeks’ Gestation and Pregnancy Complications. Fetal Diagnosis and Therapy 30: 250–265. 126. Syngelaki, A., F. E. Bredaki, E. Vaikousi, N. Maiz and K. H. Nicolaides (2011). "Body mass index at 11-13 weeks' gestation and pregnancy complications." Fetal Diagnosis and Therapy 30(4): 250-265. 127. Thuot, M., M. A. Coursol, S. Nguyen, V. Lacasse-Guay, M. F. Beauchesne, A. Fillion, A. Forget, F. Z. Kettani and L. Blais (2013). "Impact of obesity on perinatal outcomes among asthmatic women." Canadian Respiratory Journal 20(5): 345-350. 128. Vahratian, A., V. K. Misra, S. Trudeau and D. P. Misra (2010). "Prepregnancy body mass index and gestational age-dependent changes in lipid levels during pregnancy." Obstetrics and Gynecology 116(1): 107-113. 129. Vrijkotte, T. G. M., N. Krukziener, B. A. Hutten, K. C. Vollebregt, M. Van Eijsden and M. B. Twickler (2012). "Maternal lipid profile during early pregnancy and pregnancy complications and outcomes: The ABCD study." Journal of Clinical Endocrinology and Metabolism 97(11): 3917-3925. 130. Wang, T., J. Zhang, X. Lu, W. Xi and Z. Li (2011). "Maternal early pregnancy body mass index and risk of preterm birth." Archives of Gynecology and Obstetrics 284(4): 813-819. 131. Wataba, K., et al. (2006). "Impact of prepregnant body mass index and maternal weight gain on the risk of pregnancy complications in Japanese women." Acta Obstetricia et Gynecologica Scandinavica 85(3): 269-276. 132. Weiss JL, Malone FD, Emig D, Ball RH, Nyberg DA, Comstock CH, et al. Obesity, obstetric complications and cesarean delivery rate--a population-based screening study. Am J Obstet Gynecol 2004;190:1091-7. 133. Witteveen, Tom, et al. "Overweight and severe acute maternal morbidity in a low-risk pregnant population in the Netherlands." (2013): e74494. 134. Yao, R., C. V. Ananth, B. Y. Park, L. Pereira, L. A. Plante and C. Perinatal Research (2014). "Obesity and the risk of stillbirth: a population-based cohort study." American Journal of Obstetrics & Gynecology 210(5): 457.e451-459. 135. Yekta, Z., H. Ayatollahi, R. Porali and A. Farzin (2006). "The effect of pre-pregnancy body mass index and gestational weight gain on pregnancy outcomes in urban care settings in Urmia-Iran." BMC Pregnancy and Childbirth 6(15). 136. Zaheri et al. The Effect of Pre Pregnancy Body Mass Index and Gestational Weight Gain on Pregnancy Outcomes. Life Science Journal 2013;10(6s) 137. Zhao, Y. N., Q. Li and Y. C. Li (2014). "Effects of body mass index and body fat percentage on gestational complications and outcomes." Journal of Obstetrics and Gynaecology Research 40(3): 705-710. 138. Rudtanasudjatum, K. and Chullapram, T. (2008) The association between obesity and the risk of cesarean delivery and other adverse pregnancy outcomes in singleton term pregnancies. Thai Journal of Obstetrics and Gynaecology, 16, 139-145. 139. Grossetti, E et al 2004 Obstetrical complications of morbid obesity. Gynecology Journal of Obstetrics and Reproductive Biology, 2004, 33(8):739-744. 140. Chereshneva M, Hinkson L, Oteng-Ntim E. The effects of booking body mass index on obstetric and neonatal outcomes in an inner city UK tertiary referral centre. Obstet Med 2008;1:88–91. 141. Bowers, D. and W. R. Cohen (1998). "Obesity and related pregnancy complications in an inner-city clinic." Journal of perinatology: official journal of the California Perinatal Association 19(3): 216-219. 142. Bergholt T, Lim LK, Jorgensen J, Robson MS. Maternal body mass index in the first trimester and risk of caesarean section in nulliparous women in spontaneous labor. Am J Obstet Gynecol 2007;196:163. e1-163.e5 143. Aviram, A., Hod, M. and Yogev, Y. (2011) Maternal obesity: Implications for pregnancy outcome and long- term risks—A link to maternal nutrition. International Journal of Gynecology & Obstetrics, 115, S6-S10. 144. Norwitz ER et al Prolonged pregnancy. When should we intervene? Clin Obstet Gynecol 2007 50(2) 547 145. Wolfe HM, Gross TL Obesity in pregnancy. Clin Obstet Gynecol 1994;37:596-604 146. Khorshid, E. A. Elbeheidy, T. A. Quinaibi, A. M. Risk of Morbid Obesity with Pregnancy. 2004. Saudi Medical Journal 25(1): 121-122. 147. Gracie SK, Lyon AW, Kehler HL, Pennell CE, Dolan SM, McNeil DA, Siever JE, McDonald SW, Bocking AD, Lye SJ, Hegadoren KM, Olson DM, Tough S: All Our babies cohort study: recruitment of a cohort to predict women at risk of preterm birth through the examination of gene expression profiles and the environment. BMC Pregnancy Childbirth 2010, 10:87. 148. Djelantik, A.A.; Kunst, A.E.; van der Wal, M.F.; Smit, H.A.; Vrijkotte, T.G (2013). “Contribution of Overweight and Obesity to the Occurrence of Adverse Pregnancy Outcomes in a Multiethnic Cohort: Population Attributive Fractions for Amsterdam” Obstetric Anesthesia Digest, 33(1):43-45. 149. Kobayashi, Namiko, and Boon H. Lim. "Induction of labour and intrapartum care in obese women." Best Practice & Research Clinical Obstetrics & Gynaecology 29.3 (2015): 394-405. 150. Satpathy, H. K., A. Fleming, D. Frey, M. Barsoom, C. Satpathy and J. Khandalavala (2008). "Maternal obesity and pregnancy." Postgraduate medicine 120(3): E01-09. 151. Bogaerts, A., et al. (2013). "Obesity in pregnancy: Altered onset and progression of labour." Midwifery 29(12): 1303. 152. Castro, L. C. and R. L. Avina (2002). "Maternal obesity and pregnancy outcomes." Current Opinion in Obstetrics and Gynecology 14(6): 601-606. 153. Catalano PM, Ehrenberg HM: The short- and long-term implications of maternal obesity on the mother and her offspring. BJOG: Int J Obstet Gynaecol 2006, 113(10):1126–1133. 154. Caughey et al Postterm pregnancy: how can we improve outcomes? Obstetrical Gynecol Survey 2008 Nov: 63:715-24 155. Linne Y. Effects of obesity on women's reproduction and complications during pregnancy. Obes Rev. 2004 Aug;5(3):137-43 156. Nuthalapaty, F. S. and D. J. Rouse (2004). "The impact of obesity on obstetrical practice and outcome." Clinical obstetrics and gynecology 47(4): 898-913. 157. Vasudevan, C., M. Renfrew and W. McGuire (2011). "Fetal and perinatal consequences of maternal obesity." Archives of Disease in Childhood-Fetal and Neonatal Edition 96(5): F378-F382. 158. Walker, N. and J. H. Gan (2015). "Prolonged pregnancy." Obstetrics, Gynaecology and Reproductive Medicine 25(3): 83-87. 159. Wolfe, H. (1998) High prepregnancy body-mass index— A maternal-fetal risk factor. The New England Journal of Medicine, 338, 191-192. 160. Stone, R. A., J. Huffman, N. Istwan, C. Desch, D. Rhea, G. Stanziano and S. Joy (2011). "Pregnancy outcomes following bariatric surgery." Journal of Women's Health 20(9): 1363-1366. 161. Roopnarinsingh (1999) Obstetric hazards of maternal obesity. Journal of Obstetrics and Gynaecology Vol. 19, No. 5, 474-476 162. Son, G. H., J. H. Kim, J. Y. Kwon, Y. H. Kim and Y. W. Park (2013). "Risk factors for cesarean delivery after induction of labor in nulliparous women with an unfavorable cervix at or beyond 41 weeks of gestation." Gynecologic and Obstetric Investigation 76(4): 254-259. 163. Graves, B. W., S. A. DeJoy, A. Heath and P. Pekow (2006). "Maternal Body Mass Index, Delivery Route, and Induction of Labor in a Midwifery Caseload." Journal of Midwifery and Women's Health 51(4): 254-259. 164. Mandal, D., S. Manda, A. Rakshi, R. P. Dey, S. C. Biswas and A. Banerjee (2011). "Maternal obesity and pregnancy outcome: a prospective analysis." The Journal of the Association of Physicians of India 59: 486-489. 165. Michlin, R., M. Oettinger, M. Odeh, S. Khoury, E. Ophir, M. Barak, M. Wolfson and A. Strulov (2000). "Maternal obesity and pregnancy outcome." The Israel Medical Association journal: IMAJ 2(1): 10-13. 166. Suidan, R. S., J. J. Apuzzio and S. F. Williams (2012). "Obesity, comorbidities, and the cesarean delivery rate." American Journal of Perinatology 29(8): 623-627. 167. Tosson, Madiha M., and Tarek K. Alhussaini. "The impact of maternal obesity on pregnancy outcome at Assuit University Hospital." Ass Univ Bull Env Res 8.2 (2005): 1-11. 168. Oberg et al (2013) Maternal and Fetal Genetic Contributions to Postterm Birth: Familial Clustering in a Population-Based Sample of 475,429 Swedish Births. American Journal of Epidemiology. DOI: 10.1093/aje/kws244 169. Sukalich S, Mingione MJ, Glantz JC. Obstetric outcomes in overweight and obese adolescents. Am J Obstet Gynecol2006;195:851-5 170. Ritho, M.M.K., 2012. Effect of body mass index on pregnancy outcome at Kenyatta National Hospital: Cohort Study (Doctoral dissertation, University of Nairobi, Kenya). | |

**Table S3: Details of included studies as reported in the original papers**

| **Author, publication year, region, country** | **Methodology** | **Number of participants: number of cases^1^** | **Study period** | **Assessment of weight status** | **Assessment of gestational age** | **Gestational age groups (weeks)** | **BMI (kg/m^2^)**  **or weight category** | **Participants per BMI category** | **Cases per BMI category** | **Crude analysis (OR and 95% CI unless otherwise specified)** | **Adjusted analysis (OR and 95% CI unless otherwise specified)** | **Variables included in adjusted analyses** |
| --- | --- | --- | --- | --- | --- | --- | --- | --- | --- | --- | --- | --- |
| Abenhaim et al 2007^16^  Canada | Retrospective Cohort | 18,633:not reported | 04/1987 -03/1997 | Pre-pregnancy BMI, method of assessment not reported^#^ | Not reported | >37-42 *  >42 | 20-24.9 ^+^  <19.9  25-29.9  30-39.9  >40 | 10,015  4,310  3,067  1,137  104 | Not reported | Not reported | 1(1)  1.07 (0.86-1.33)  1.13 (0.89-1.45)  0.84 (0.55-1.28)  0.76 (0.19-3.10) | Maternal Age  Parity  Smoking  Pre-existing diabetes |
| Al-Rayyan et al 2010^17^  Jordan | Retrospective Cohort | 901:109 | 01/1990 - 12/2000 | Early pregnancy measured BMI | Not reported | 37-41 *  >42 | <30 ^+^  ≥30.0 | 461  440 | 55  54 | Not reported | Not reported | N/A |
| Arora et al 2013^18^  Thailand | Retrospective Cohort | 4,764:32 | 02/2011 - 08/2012 | Pre-pregnancy BMI, method of assessment not reported^#^ | Not reported | 37-41 *  42 | 18.5-24.9^+^  <18.5  25-29.9  ≥30 | 3,129  912  535  188 | 19  7  5  1 | Not reported | Not reported | N/A |
| Arrowsmith et al 2011^19^  UK | Retrospective Cohort | 20,599:Not reported | 01/2004 - 12/2008 | Early pregnancy measured BMI | USS | 37-41^+2^ *  41^+3^ | 20-24.9^+^  <19.9  25-29.9  30-34.9  35-39.9  >40 | Not reported | Not reported | Not reported | 1(1)  0.75 (0.66-0.85)  1.24 (1.14-1.34)  1.52 (1.37-1.70)  1.75 (1.48-2.07)  2.27 (1.78-2.86) | Maternal age  Race  Parity  Hypertension  Diabetes mellitus  Smoking |
| Basu et al 2010^20^ South Africa | Retrospective Cohort | 738:132 | 02/2006 and 09/2006 | Early pregnancy measured BMI | USS + LMP | 37-41 *  >41 | 18.5-24.9^+^  25-29.9  30-39.9  >40 | 139  273  288  38 | 18  54  49  11 | Not reported | Not reported | N/A |
| Bhattacharya 2007^21^  UK | Retrospective Cohort | 21,511:1,374 | 1976-2005 | Early pregnancy measured BMI | USS + LMP | 37-41 *  >41 | 20-24.9^+^  <19.9  25-29.9  30-34.9  >35 | 12,539  2,497  4,735  1,615  125 | 773  108  350  136  7 | 1 (1)  0.7 (0.6-0.8)  1.2 (1.1-1.3)  1.4 (1.1-1.6)  0.8 (0.4-1.7) | 1 (1)  0.9 (0.7-1.1)  0.9 (0.8-1.1)  0.9 (0.7-1.1)  0.8 (0.4-1.8) | Relevant sociodemographic characteristics  Year of delivery. |
| Briese et al 2011^22^  Germany | Retrospective Cohort | Not reported | 1998-2000 | BMI, method of assessment not reported | Not reported | Not reported | 18.5-24.9  ≥30 | Not reported | Not reported | Not reported | 1 (1)  1.45 (1.38-1.52) | Age  Smoking status  Single mother status  Education  Only included primiparous women |
| Caughey et al 2009^23^  USA | Retrospective Cohort | Not reported | 01/1995 -12/1999 | BMI, method of assessment not reported | Not reported | 37-<41*  ≥41  37-<42*  ≥42 | Not obese ^+^  Obese (BMI not defined) | Not reported | Not reported | Not reported | 1 (1)  1.29 (1.18, 1.40)  1 (1)  1.20 (0.99, 1.46) | Excluding chronic hypertension, diabetes mellitus, and gestational diabetes mellitus.  Controlling for paternal  race/ethnicity. |
| Cedergren 2004^24^  Sweden | Retrospective Cohort | 580,970:  45,288 | 01/1992 - 12/2001 | Early pregnancy measured BMI | USS | 37-41^+6^ *  ≥42 | 19.8-26^+^  29.1-35  35.1-40  >40 | 501,954  64,286  11,605  3,125 | 37,640  6,072  1,197  379 | Not reported | 1 (1)  1.37 (1.33-1.41)  1.49 (1.40-1.58)  1.80 (1.62-2.01) | Maternal age  Parity  Smoking  Year of birth |
| Denison et al 2008^25^  Sweden | Retrospective Cohort | 143,519:9,759 | 1998-2002 | Early pregnancy measured BMI | USS + LMP | 37-41^+6^ *  ≥42 | 20-25^+^  <20  25-<30  30-<35  ≥35 | 84,963  18,227  30,139  7,463  2,440 | Not reported | Term median BMI 22.9 (IQR 21.0–25.3)  Postdate median BMI 23.4 (IQR 21.5–26.0)  p<0.0001 | Not reported | N/A |
| El-Gilany and Hammad 2010^26^  Saudi Arabia | Prospective Cohort | 787:22 | 01/2007-12/2007 | Early pregnancy measured BMI | LMP | 37-42 *  >42 | 18.5-24.9^+^  <18.5  25-29.9  ≥30 | 307  67  187  226 | 4  2  5  11 | RR (95% CI)  1 (1)  2.3 (0.4-12.3)  2.0 (0.6-7.1)  3.7 (1.2-11.6) | Not reported | N/A |
| Halloran et al 2012^27^  USA | Retrospective Cohort | 267,126:  51,420  267,126:  13,392 | 2000-2006 | Self-report pre-pregnancy BMI | Clinical measurement | 37-40 *  =41  =42 | 18.5-24.9^+^  <18.5  25-29.9  ≥30  18.5-24.9^+^  <18.5  25-29.9  ≥30 | 180,056  19,354  76,792  62,924  180,056  19,354  76,792  62,924 | 26,487  2,783  11,794  10,356  6,866  853  2,997  2,676 | Not reported | Not reported | N/A |
| Johnson et al 1992^28^  USA | Retrospective Cohort | 3,203:314 | 01/1987-12/1989 | Self-report pre-pregnancy BMI | USS | 38-42 *  >42 | <19.8^+^  19.8-26  27-29  >29 | 755  1,633  329  486 | 61  157  40  56 | 1 (1)  1.22 (0.89-1.66)  1.58 (1.03-2.4)  1.49 (1.01-2.2) | Not reported | N/A |
| Khashan and Kenny 2009^29^ UK | Retrospective Cohort | 89,513:4,706 | 01/2004-12/2006 | Early pregnancy measured BMI | USS + LMP | Not reported*  ≥41 | 18.5-24.9^+^  <18.5  25-29.9  30-40  >40 | 42,147  2325  23,757  13,386  1,543 | 2,213  99  1,404  897  93 | 1 (1)  0.79 (0.65-0.96)  1.13 (1.06-1.21)  1.28 (1.19-1.38)  1.17 (0.95-1.43) | 1 (1)  0.81 (0.67-0.99)  1.17 (1.09-1.25)  1.35 (1.25-1.45)  1.24 (1.02-1.52) | Maternal age  Parity Race |
| Kistka et al 2007^30^  USA | Retrospective Cohort | 368,633:Not reported | 1989-1997 | BMI, method of assessment not reported | Not reported | 37-41+6 *  ≥42 | Reference not defined^+^  <20  >35 | Not reported | Not reported | 1 (1)  0.90 (0.88-0.93)  1.25 (1.19-1.32) | 1 (1)  0.85 (0.82-0.87)  1.23 (1.16-1.29) | Socioeconomic status  Maternal medical risk factors  Year of delivery |
| Kitiyodom and Tongswatwong 2008^31^  Thailand | Retrospective Cohort | 1,350:166 | 10/2004-09/2006 | Early pregnancy measured BMI | USS + LMP | Reference not defined^+^  Post-term not defined | 20-24.9^+^  >25 | 1,020  330 | 110  56 | 1 (1)  1.7 (1.19-2.44) | Not reported | N/A |
| Knight et al 2010^32^  UK | Prospective Cohort | 1,280:56 | 09/2007-08/2008 | Pregnancy measured BMI | Not reported | Reference not defined ^+^  >42 | <50^+^  ≥50 | 630  650 | 24  32 | 1 (1)  1.31 (0.76-2.25) | 1 (1)  1.35 (0.77-2.37) | Maternal age  Parity  Socioeconomic status  Ethnicity  Smoking |
| Konje et al 1993^33^  UK | Case Control | 760:14 | 01/1989-06/1990 | Early pregnancy measured BMI | USS | 37-42 *  >42 | 17-24^+^  30.4-53.0 | 299  461 | 11  3 | Not reported | Not reported | N/A |
| Leung et al 2008^34^  Hong Kong | Retrospective Cohort | 27,372:4,118 | 01/1995-12/2005 | Early pregnancy BMI | Not reported | 37-40+6 *  ≥41 | 18.5-<23^+^  <18.5  ≥23-<25  ≥25-<27.5  ≥27.5-<30  ≥30 | 16,303  2,434  4,346  2,617  1,062  610 | 2,430  322  654  429  175  108 | Not reported | 1 (1)  0.84 (0.74-0.95)  1.06 (0.97-1.17)  1.21 (1.08-1.36)  1.25 (1.05-1.48)  1.34 (1.09-1.66) | Only included Chinese ethnicity. Adjusted for confounding factors, not specified. |
| Lumme et al 1995^35^  Finland | Prospective Cohort | 8,719:439 | 07/1985-06/1986 | Early pregnancy measured BMI | Not reported | 37-41 *  >41 | 19-24.9^+^  <19  25-29.9  ≥30 | 1,037  6,173  1,177  332 | 136  228  63  12 | Not reported | 1 (1)  1.0 (0.7-1.4)  1.6 (1.2-2.1)  1.1 (0.6-1.9) | Maternal age  Parity  Education  Smoking  Diabetic and Hypertensive complications |
| Mancuso et al 1991^36^  Italy | Case Control | 138:4 | Not reported | Self-report pre-pregnancy BMI | Not reported | 38-41 *  >42 | 15.2-26.6^+^  >30 | 82  56 | 1  3 | Not reported | Not reported | N/A |
| Manzanares et al 2012^37^  Spain | Case Control | 2,714:196 | 2007-2009 | Early pregnancy measured BMI | Not reported | 37-41^+2^ *  >41^+3^ | 18.5-25^+^  <18.5  >35 | 2,341  147  226 | 174  12  10 | Not reported | 1 (1)  0.81 (0.35-1.91)  0.72 (0.34-1.55) | Maternal age  Parity  Hypertension  Diabetes |
| Morgan et al 2014^38^  UK | Prospective Cohort | 440:28 | 11/2010-02/2013 | Pre-pregnancy BMI, method of assessment not reported^#^ | USS | Reference not defined ^+^ =42 | 18.5-24.9^+^  25-29.9  >29.9 | 234  206 | 10  18 | 1 (1)  2.18 (0.99-4.84) | Not reported | N/A |
| Navid et al 2013^39^  Pakistan | Case Control | 200:57 | 05/2011 -07/2012 | Early pregnancy BMI, method of assessment not reported | USS | 37-40*  >40 | 18-24.9^+^  25-35 | 100  100 | 25  32 | Not reported | Not reported | N/A |
| Nohr et al 2009^40^  Denmark | Prospective Cohort | 4,700:1,541 | 1996-2002 | Self-reported pre-pregnancy BMI | Not reported | 37-41*  >41 | 15-33.3^+^  32.6-<35  35-<37.5  ≥37.5 | 2,354  853  721  770 | 688  292  277  283 | Not reported | 1 (1)  1.3 (1.1-1.5)  1.5 (1.3-1.8)  1.4 (1.2-1.7) | Age  Parity  Maternal height  Smoking  Alcohol consumption  Physical exercise  Social group |
| Olesen et al 2006^41^  Denmark | Retrospective Cohort | 47,338:5,227 | 1996-2004 | BMI –self reported | LMP + USS | 37- 41^+6^ *  ≥42 | 20-24^+^  <20  25-29  30-34  ≥35 | 26,486  7,918  9,201  2,713  1,020 | 2,800  736  1,165  374  152 | 1  0.87  1.23  1.35  1.48  95%CI not reported | 1 (1)  0.87 (0.80-0.94)  1.24 (1.15-1.34)  1.37 (1.22-1.54)  1.52 (1.28-1.82) | Maternal age  Parity |
| Raatikainen et al 2006^42^  Finland | Retrospective Cohort | 25,601:1,233 | 01/1989 -12/2001 | Early pregnancy measured BMI | Not reported | Reference not defined ^+^  >42 | ≤25^+^  26-29  ≥30 | 20,333  3,388  1,880 | 935  193  105 | Not reported | Not reported | N/A |
| Robinson et al 2005^43^  Canada | Retrospective Cohort | 84,055:5,689 | 01/1988 -12/1992 | Pre-pregnancy BMI, method of assessment not reported^#^ | Not reported | Reference not defined ^+^ >41 | 55-75Kg^+^  ≥90-120Kg  >120Kg | 74,566  8,774  715 | 4,997  647  45 | 1 (1)  1.10 (1.01-1.20)  0.91 (0.67-1.23) | 1 (1)  1.18 (1.08-1.28)  0.99 (0.74-1.34) | Maternal age  Marital status  Parity  Smoking Socioeconomic status |
| Rode et al 2005^44^  Denmark | Prospective Cohort | 8,463:Not reported | 1998 - 2001 | Self-report pre-pregnancy BMI | USS | 37-42*  >42 | <25^+^  25-29.9  ≥30 | Not reported | Not reported | Not reported | 1 (1)  1.4 (1.2-1.7)  1.4 (1.1-1.9) | Maternal age  Smoking  Ethnic background  Type of conception |
| Roos et al 2010^45^  Sweden | Retrospective Cohort | 972,883: 85,488 | 01/1992 -12/2006 | Early pregnancy measured BMI | USS + LMP | 37-41+6* ≥42 | 20-24.9^+^  <20  25-29.9  ≥30 | 560,667  105,643  224,550  82,013 | 46,323  6,486  22,834  9,845 | Not reported | 1 (1)  0.74 (0.72-0.76)  1.31 (1.29-1.33)  1.63 (1.59-1.67) | Maternal age  Parity  Education  BMI  Smoking  Country of origin  Mother living with father |
| Schrauwers and Dekker 2009^46^  Australia | Retrospective Cohort | 348:3 | 01/2006 -06/2006 | Early pregnancy BMI, method of assessment not reported | Not reported | 37-41*  >41 | 19.1-25^+^  25.1-30  30.1-40  >40 | 93  92  108  55 | 0  1  2  0 | Not reported | Not reported | N/A |
| Scott-Pillai et al 2013 ^47^  UK | Retrospective Cohort | 30,298:Not reported | 2004-2011 | Early pregnancy measured BMI | Not reported | Reference not defined ^+^ >41 | 18.5-24.99^+^  <18.50  25-29.99  30-34.99  35-39.99  ≥40 | Not reported | Not reported | Not reported | 1 (1)  0.5 (0.2-1.0)  0.9 (0.7-1.1)  0.8 (0.5-1.1)  0.9 (0.5-1.6)  0.8 (0.4-1.7) | Age  Parity Year of birth  Social deprivation  Smoking |
| Sharief and Tarik 2000^48^  Iraq | Prospective Cohort | 40:6 | 12/1997-08/1998 | Early pregnancy measured weight | Not reported | Reference not defined ^+^ Post-term not defined | <=90Kg  >90Kg | 20  20 | 3  3 | Not reported | Not reported | N/A |
| Stotland et al 2007^49^  USA | Retrospective Cohort | 10,878:2,402  10,878:595 | 1990-2001 | Self-report pre-pregnancy BMI | USS + LMP | 37-<41*  ≥41  37-<42*  ≥42 | 19.8-26^+^  <19.8  26.1-29  >29 | 6,477  2,171  1,213  1,017  6,477  2,171  1,213  1,017 | 1,418  397  297  290  350  83  89  73 | Not reported | 1 (1)  0.83 (0.72-0.95)  1.29 (1.10-1.52)  1.81 (1.50-2.18)  1 (1)  0.78 (0.60-1.01)  1.51 (1.15-1.97)  1.69 (1.23-2.31) | Maternal age  Ethnicity  Parity  Gestational weight gain  Insurance status  Hypertension  Diabetes  Smoking |
| Usha Kiran et al 2005^50^  UK | Retrospective Cohort | 8,350:2,768 | 1990-1999 | Early pregnancy measured BMI | USS | 37-41*  >41 | 20-30^+^  >30 | 7,673  677 | 2,490  278 | 1 (1)  1.4 (1.2-1.7) | Not reported | N/A |
| Vaswani and Balachandran 2013^51^  United Arab Emirates | Retrospective Cohort | 1,985:147 | 12/2010 -10/2011 | Early pregnancy measured BMI | Not reported | 37-41*  >41 | 18.5-24.9^+^  25-29.9  30-34.9  35-39.9  ≥40 | 420  635  520  280  130 | 20  48  43  24  12 | Not reported | 1 (1)  1.54 (0.89-2.65)  1.69 (0.96-2.98)  1.78 (0.93-3.42)  2.99 (1.35-6.65) | Age  Parity  Hypertension Diabetes |
| Vinturache et al 2014^52^  Canada | Prospective Cohort | 1,996:5 | 05/2008 -12/2010 | Self-report pre-pregnancy BMI | Not reported | 37-41^+6^ *  ≥42 | 18.5-24.99^+^  25-29.99  ≥30 | 1,313  472  211 | 4  1  0 | Not reported | Not reported | N/A |
| Voigt et al 2008^53^  Germany | Retrospective Cohort | 303,960: 34,266 | 1998-2000 | Pre-pregnancy BMI, method of assessment not reported^#^ | Not reported | Term, not defined ^+^  Post-term, not defined | 18.5-24.99^+^  40-44.99  ≥45 | 300,299  2,946  715 | 33,703  452  111 | Not reported | Not reported | N/A |
| Yazdani et al 2006^54^  Iran | Retrospective Cohort | 966:33 | 2008-2009 | Early pregnancy BMI, method of assessment not reported | USS + LMP | Term, not defined ^+^  Post-term, not defined | 20-24.9^+^  ≤19.9  25-29.9  30-34.9  >35 | 403  126  340  92  5 | 14  4  12  3  0 | Not reported | Not reported | N/A |

Footnote: ^1^ In studies which presented data for preterm, term and post-term births, the numbers of participants and cases were calculated following exclusion of pre-term births when possible. ^#^Weight assessment is presumed to be self-reported as pre-pregnancy BMI was used, although this was not explicitly stated. ^+^ Reference group for BMI. * Reference group for gestational age. Abbreviations: BMI = body mass index; CI = confidence interval; IQR = inter quartile range; LMP = last menstrual period; N/A = not applicable; OR = odds ratio; RR = relative risk; USS = ultrasound scan

**Table S4: Contacting authors for additional information**

| **Paper** | **Reason For Contacting** | **Original Data**  (BMI Kg/m^2^) | **Original Data**  **(**Participants:Cases) | **Author Response^#^** | **Definitions Provided** | **Data Provided**  (BMI Groups) | **Data Provided**  (Participants:Cases) |
| --- | --- | --- | --- | --- | --- | --- | --- |
| Abenhaim et al 2007^16^ | - To provide frequencies for cases^ - Split BMI 30-39.9 into WHO obesity classes I and II | 20-24.9  <19.9  25-29.9  30-39.9  >40 | 10,015:not reported  4,310:not reported  3,067:not reported  1,137:not reported  104:not reported | Unable to provide data | N/A | N/A | Not provided^\|^ |
| Al-Rayyan et al 2010^17^ | - To provide frequencies according to specified WHO BMI categories^ | <30  >30 | 461:55  440:54 | No response | N/A | N/A | Not provided^\|^ |
| Arora et al 2013^18^ | - To provide frequencies for WHO BMI categories >30 | >30 | 188:1 | Additional data provided | N/A | 30-34.9  35-39.9  ≥40 | 159:0  35:0  9:1 |
| Arrowsmith et al 2011^19^ | - To provide frequencies for participants and cases^ | 20-24.9  <19.9  25-29.9  30-34.9  35-39.9  >40 | Not reported | Additional data provided | N/A | 20-24.9^+^  <19.9  25-29.9  30-34.9  35-39.9  >40 | 9,374:2,193  2,002:367  5,262:1,428  2,028:634  697:234  298:123 |
| Basu et al 2010^20^ | - Split BMI 30-39.9 into WHO obesity classes I and II | 30-39.9 | 298:49 | No response | N/A | N/A | Not provided |
| Bhattacharya et al 2007^21^ | - Definition used for comparison group for gestational age | N/A | N/A | Definition provided | 37-42 | N/A | N/A |
| Briese et al 2011^22^ | - Definition for gestational age reference group - Definition used for post-term - To provide frequencies for participants and cases^ | 18.5-24.9  ≥30 | Not reported | Provided definitions. Unable to provide frequencies. | >37-<42  ≥42 | N/A | Not provided^\|^ |
| Caughey et al 2009^23^ | - To provide frequencies according to specified WHO BMI categories^ | Not reported | Not reported | Unable to provide data | N/A | Not provided | Not provided^\|^ |
| Cedergren 2004^24^ | - To provide frequencies the BMI category >26-29. | 19.8-26  29.1-35  35.1-40  >40 | 501,954:37,640  64,286:6,072  11,605:1,197  3,125:379 | Additional data provided | N/A | 26.1-29 | 95,675:8,381 |
| Denison et al 2008^25^ | - To provide frequencies according to specified WHO BMI categories^ | 20-24.9  <20  25-29.9  30-34.9  ≥35 | Not reported | Unable to provide data | N/A | N/A | Not provided^\|^ |
| El-Gilany and Hammad 2010^26^ | - Split BMI ≥30 into WHO obesity classes | ≥30 | 226:11 | Additional data provided | N/A | 30-34.9  35-39.9  ≥40 | 138:1  59:4  29:6 |
| Halloran et al 2012^27^ | - Split BMI ≥30 into WHO obesity classes for 41 and 42 weeks gestation | **41**  ≥30  **42**  ≥30 | 62,924:10,356  62,924:2,676 | Additional data provided | N/A | **41**  30-34.9  35-39.9  ≥40  **42**  30-34.9  35-39.9  ≥40 | 35,767:5,832  16,802:2,741  10,355:1,777  35,767:1,491  16,802:743  10,355:442 |
| Johnson et al 1992^28^ | - Split BMI >29 into WHO obesity classes | >29 | 486:56 | Unable to provide data | N/A | N/A | Not provided |
| Khashan and Kenny 2009^29^ | - Split BMI 30-40 into WHO obesity classes - Definition of gestational age reference group | 30-40 | 12,489:897 | Additional data provided | 37-<42 | 30-34.9  35-39.9 | 9,983:650  3,574:247 |
| Kistka et al 2007^30^ | - Definition of BMI reference group - To provide frequency data^ | Reference group not reported  <20  >35 | Not reported | Unable to provide data | N/A | N/A | Not provided^\|^ |
| Kitiyodom and Tongswatwong 2008^31^ | - Split BMI >25 into WHO categories^ - Definition of gestational age reference group and post-term | >25 | 330:56 | Additional data provided | 37-<42  ≥42 | 25-29.9  30-34.9  35-39.9  ≥40 | 211:25  52:24  21:6  2:1 |
| Knight et al 2010^32^ | - Split BMI <50 into WHO categories^ - Definition of gestational age reference group | <50 | 630:24 | Additional data provided | 37-<42 | 18.5-24.9^+^  <18.5  25-29.9  30-34.9  35-39.9  ≥40 | 267:12  15:0  173:7  77:3  28:2  11:0 |
| Konje et al 1993^33^ | - To provide frequencies for specific BMI groups >25 | Not reported | 299:11  461:3 | Unable to provide data | N/A | Not provided | Not provided |
| Leung et al 2008^34^ | - To provide frequencies for specific BMI groups >30 | ≥30 | 610:108 | No response | N/A | N/A | Not provided |
| Lumme et al 1995^35^ | - To provide frequencies for specific BMI groups >30 | ≥30 | 332:12 | No response | N/A | N/A | Not provided |
| Mancuso et al 1991^36^ | - To provide frequencies according to specified WHO BMI categories^ | 15.2-26.6  >30 | 82:1  56:3 | No response | N/A | N/A | Not provided^\|^ |
| Manzanares et al 2012^37^ | - To provide frequencies for BMI groups 25-29.9, 30-34.9, 35-39.9, ≥40 | 18.5-25  >35 | 2,341:174  226:10 | Additional data provided | N/A | 25-29.9  30-34.9  35-39.9  ≥40 | 914:64  321:19  129:6  86:4 |
| Morgan et al 2014^38^ | - To provide frequencies for BMI groups 25-29.9 - Split >29.9 into WHO obesity classes - Definition for gestational age reference group. | 18.5-24.9  >29.9 | 234:10  206:18 | Provided definition for gestational age. Unable to provide frequency data | 37-<42 | N/A | Not provided |
| Navid et al 2013^39^ | - Split BMI group 25-35^ | 18-24.9^+^  25-35 | 100:25  100:32 | No response | N/A | N/A | Not provided^\|^ |
| Nohr et al 2009^40^ | - To provide frequencies according to specified WHO BMI categories^ | 15-33.3  32.6-<35  35-<37.5  ≥37.5 | 2,354:688  853:292  721:277  770:283 | Additional data provided | N/A | 18.5-24.9  <18.5  25-29.9  30-34.9  35-39.9  ≥40 | 1,261:71  87:3  332:17  677:40  731:50  272:22 |
| Olesen et al 2006^41^ | - Split BMI ≥35 | ≥35 | 1,020:154 | Unable to provide data | N/A | N/A | Not provided |
| Raatikainen et al 2006^42^ | - Split BMI ≥30 into obesity classes I-III - Definition for gestational age reference group | ≤25  26-29  ≥30 | 20,333:935  3,388:193  1,880:105 | No response | Not provided* | Not provided | Not provided |
| Rode et al 2005^44^ | - To provide frequencies of participants and cases according to specified WHO BMI categories^ | <25^+^  25-29.9  ≥30 | Not reported | Additional data provided | N/A | 18.5-24.9  <18.5  25-29.9  30-34.9  35-39.9  ≥40 | 5,986:567  364:25  1,298:162  326:39  81:12  37:3 |
| Roos et al 2010^45^ | - Split BMI ≥30 into obesity classes I-III | ≥30 | 82,013:9,845 | Unable to provide data | N/A | Not provided | Not provided |
| Scott-Pillai et al 2013^47^ | - To provide frequencies of participants and cases^ - Definition for gestational age reference group | 18.5-24.99  <18.50  25-29.99  30-34.99  35-39.99  ≥40 | Not reported | Additional data provided | >37-41 | 18.5-24.99  <18.50  25-29.99  30-34.99  35-39.99  ≥40 | 15,046:3,261  803:149  7,917:1,776  3,120:667  1,121:238  541:121 |
| Stotland et al 2007^49^ | - To split BMI groups >29 into obesity classes I-III for both 41 and 42 weeks gestation | **41**  >29  **42**  >29 | 1,017:290  1,017:73 | Unable to provide data | N/A | N/A | Not provided |
| Usha Kiran et al 2005^50^ | - To split BMI groups 20-30 and >30 according to WHO BMI categories^ | 20-30  >30 | 7,673:2,490  677:278 | Unable to provide data | N/A | N/A | Not provided^\|^ |
| Voigt et al 2008^53^ | - To provide frequencies for WHO categories between 25 and 39.9 - Definitions for gestational age reference group and post-term | 18.5-24.99^+^  40-44.99  ≥45 | 300,299:33,703  2,946:452  715:11 | Provided definitions. Unable to provide frequencies. | 37-<42  ≥42 | Not provided | Not provided |
| Yazdani et al 2006^54^ | - Definitions for gestational age reference group and post-term | N/A | N/A | No response | Not provided* | N/A | N/A |

Footnote: # Non-responding authors contacted up to three times

^ Essential data request for inclusion in meta-analysis

^|^ Excluded from meta-analysis after contacting author, due to lack of essential data,

* Assumptions made as per methods section of manuscript.

**Table S5: Quality scores for all included studies**

| **Paper** | **Newcastle Ottawa Scale Question number and score allocated** | | | | | | | | **Independent reviewer initials** |
| --- | --- | --- | --- | --- | --- | --- | --- | --- | --- |
|  | **1** | **2** | **3** | **4** | **5** | **6** | **7** | **Total Stars** |  |
| Abenhaim et al 2007 ^16^ | a ***** | a ***** | c | b ***** | d | b | c | 3 | LC & LH |
| Al-Rayyan et al 2010 ^17^ | d | a ***** | a ***** | c | d | b | d | 2 | LC & NH |
| Arora et al 2013 ^18^ | b ***** | a ***** | d | c | d | b | b ***** | 3 | NH & LH |
| Arrowsmith et al 2011 ^19^ | a ***** | a ***** | a ***** | a&b****** | b ***** | a ***** | b ***** | 8 | LC & NH |
| Basu et al 2010 ^20^ | b ***** | a ***** | d | c | b ***** | b | d | 3 | NH & LH |
| Bhattacharya et al 2007 ^21^ | c | a ***** | a ***** | b ***** | b ***** | b | b ***** | 5 | JR & LH |
| Briese et al 2011^22^ | a ***** | a ***** | d | b ***** | d | b | a ***** | 4 | LC & NH |
| Caughey et al 2009 ^23^ | a ***** | a ***** | d | b ***** | d | a ***** | d | 4 | LH & NH |
| Cedergren 2004 ^24^ | a ***** | a ***** | d | b ***** | b ***** | b | b ***** | 5 | NH & LH |
| Denison et al 2008 ^25^ | a ***** | a ***** | a ***** | c | b ***** | a ***** | c | 5 | LC & LH |
| El Gilany & Hammad 2010 ^26^ | a ***** | a ***** | a ***** | c | c |  | c | 3 | LC & LH |
| Halloran et al 2012 ^27^ | a ***** | a ***** | c | b ***** | b ***** | b | b ***** | 5 | LC & NH |
| Johnson et al 1992 ^28^ | b ***** | a ***** | c | b ***** | b ***** | b | b ***** | 5 | LC & NH |
| Khashan & Kenny 2009 ^29^ | a ***** | a ***** | a ***** | b ***** | b ***** | b | c | 5 | LC & NH |
| Kistka et al 2007 ^30^ | c | a ***** | d | b ***** | b ***** | b | b ***** | 4 | LH & JR |
| Kitiyodom & Tongswatwong 2008 ^31^ | d | a ***** | a ***** | c | b ***** | b | d | 3 | NH & RV |
| Knight et al 2010 ^32^ | b ***** | a ***** | d | b ***** | d | b | b ***** | 4 | LC & NH |
| Konje et al 1993 ^33^ | c | a ***** | a ***** | b ***** | b ***** | b | d | 4 | NH & LH |
| Leung et al 2008 ^34^ | a ***** | a ***** | d | b ***** | d | b | b ***** | 4 | LC & LH |
| Lumme et al 1995 ^35^ | a ***** | a ***** | b ***** | c | d | b | b ***** | 4 | LC & NH |
| Mancuso et al 1991 ^36^ | d | a ***** | d | c | d | b | d | 1 | LC & NH |
| Manzanares et al 2012 ^37^ | a ***** | a ***** | a ***** | b ***** | d | b | c | 4 | LC & NH |
| Morgan et al 2014 ^38^ | b ***** | a ***** | d | b ***** | b ***** | b | c | 4 | LC & LH |
| Navid et al 2013 ^39^ | d | a ***** | d | c | a ***** | b | d | 2 | NH & LH |
| Nohr et al 2009 ^40^ | b ***** | a ***** | c | b ***** | d | b | b ***** | 4 | NH & LH |
| Olesen et al 2006 ^41^ | b ***** | a ***** | c | b ***** | c | b | d | 3 | LH & LC |
| Raatikianen et al 2006 ^42^ | a ***** | a ***** | a ***** | b ***** | d | b | b ***** | 5 | LH & JR |
| Robinson et al 2005 ^43^ | a ***** | a ***** | d | b ***** | d | b | b ***** | 4 | NH & LH |
| Rode et al 2005 ^44^ | b ***** | a ***** | c | b ***** | a ***** | b | b ***** | 5 | NH & LC |
| Roos et al 2010 ^45^ | a ***** | a ***** | a ***** | a&b****** | b ***** | a ***** | b ***** | 8 | NH & LH |
| Schrauwers & Dekker 2009 ^46^ | b ***** | a ***** | d | c | d | b | d | 2 | LH & NH |
| Scott-Pillai et al 2013 ^47^ | a ***** | a ***** | a ***** | a&b****** | d | a ***** | b ***** | 7 | LC & NH |
| Sharief & Tarik 2000 ^48^ | c | a ***** | a ***** | b ***** | d | b | d | 3 | LH & NH |
| Stotland et al 2007 ^49^ | a ***** | a ***** | d | a&b****** | b ***** | a ***** | d | 6 | NH & JR |
| Usha Kiran et al 2004 ^50^ | c | a ***** | a ***** | c | b ***** | b | b ***** | 4 | LC & JR |
| Vaswani and Balachandran 2013 ^51^ | a ***** | a ***** | a ***** | b ***** | d | b | d | 4 | LH & NH |
| Vinturache et al 2014 ^52^ | b ***** | a ***** | c | b ***** | d | a ***** | b ***** | 5 | NH & LH |
| Voigt et al 2008 ^53^ | a ***** | a ***** | d | c | d | b | d | 2 | LC & NH |
| Yazdani et al 2012 ^54^ | c | a ***** | d | c | b ***** | b | c | 2 | NH & RV |

Footnote: Newcastle-Ottawa question numbers 1-7, answers a-d, and associated number of stars (*) are detailed in fig. S2. Minimum number of possible stars to be awarded = 0, maximum number of possible stars to be awarded = 8. Reviewers initials relate to manuscript authors JR: Judith Rankin, LC: Lisa Crowe, LH: Louise Hayes, NH: Nicola Heslehurst, and RV: Rute Vieira.

**Figure S3: Exploration of the use of adjusted or unadjusted data for post-term birth (≥ 42 weeks and ≥ 41 weeks) meta-analysis**

**Figure S3a) Association between maternal BMI and post-term birth ≥42 weeks: unadjusted data**

Overall (I

-squared = 98.2%, p = 0.000)

Stotland

et al 2007 ^49^

Roos

et al 2010 ^45^

Olesen

et al 2006 ^41^

Cedergren

et al 2004 ^24^

10878

972873

47338

676645

595

85488

5227

53669

1.21 (1.13, 1.29)

1.29 (1.16, 1.44)

1.26 (1.25, 1.27)

1.17 (1.13, 1.20)

1.15 (1.14, 1.16)

.2

.5

1

1.5

2

2.5

**Source**

**Participants**

**(n)**

**OR per 5 BMI units**

**(95% CI)**

**Post-term**

**≥42 weeks (n)**

Figure legend: Meta-analysis of the four studies which provided both unadjusted and adjusted data for maternal BMI and post-term birth, showing the overall effect size (OR) and 95% confidence interval for the association when using the unadjusted data to compute the OR for the continuous BMI. Abbreviations: OR = odds ratio; BMI = body mass index; n = number of individuals.

**Figure S3b) Association between maternal BMI and post-term birth ≥42 weeks: adjusted data**

1.24 (1.15, 1.33)

1.17 (1.13, 1.21)

1.30 (1.29, 1.31)

1.35 (1.20, 1.53)

1.18 (1.17, 1.20)

Stotland

et al 2007 ^49^

Roos

et al 2010 ^45^

Olesen

et al 2006 ^41^

Cedergren

et al 2004 ^24^

10878

972873

47338

676645

595

85488

5227

53669

Overall (I

-squared = 98.3%, p = 0.000)

**Source**

**Participants**

**(n)**

**Post-term**

**≥42 weeks (n)**

**OR per 5 BMI units**

**(95% CI)**

.2

.5

1

1.5

2

2.5

Figure legend: Meta-analysis of the four studies which provided both unadjusted and adjusted data for maternal BMI and post-term birth, showing the overall effect size (OR) and 95% confidence interval for the association when using the adjusted data to compute the OR for the continuous BMI. Abbreviations: OR = odds ratio; BMI = body mass index; n = number of individuals.

**Figure S3c) Association between maternal BMI and post-term birth ≥41 weeks: unadjusted data**

Overall (I

-

squared = 96.9%, p

<

0.00

1

)

Stotland

et

al

2007 ^49^

Vaswani

et

al

2013 ^51^

Arrowsmith

et

al

2011 ^19^

Scott

-

Pilai

et

al

2013 ^47^

10878

1985

19661

28548

2402

147

4979

6212

1.15 (1.02, 1.30)

1.16 (1.01, 1.34)

1.22 (1.15, 1.29)

1.23 (1.19, 1.27)

1.01 (0.98, 1.04)

**Source**

**Participants**

**(n)**

**OR per 5 BMI units**

**(95% CI)**

**Post-term**

**≥41 weeks (n)**

.2

.5

1

1.5

2

2.5

Figure legend: Meta-analysis of the four studies which provided both unadjusted and adjusted data for maternal BMI and post-term birth, showing the overall effect size (OR) and 95% confidence interval for the association when using the unadjusted data to compute the OR for the continuous BMI. Abbreviations: OR = odds ratio; BMI = body mass index; n = number of individuals.

**Figure S3d) Association between maternal BMI and post-term birth ≥41 weeks: adjusted data**

1.18 (1.06, 1.31)

1.22 (1.04, 1.44)

1.30 (1.22, 1.40)

1.24 (1.20, 1.28)

0.96 (0.86, 1.07)

.2

.5

1

1.5

2

2.5

Stotland

et

al

2007 ^49^

Vaswani

et

al

2013 ^51^

Arrowsmith

et

al

2011 ^19^

Scott

-

Pilai

et

al

2013 ^47^

10878

1985

19661

28548

2402

147

4979

6212

**Source**

**Participants**

**(n)**

**OR per 5 BMI units**

**(95% CI)**

**Post-term**

**≥41 weeks (n)**

Overall (I-squared = 87.3%, p<0.001)

Figure legend: Meta-analysis of the four studies which provided both unadjusted and adjusted data for maternal BMI and post-term birth, showing the overall effect size (OR) and 95% confidence interval for the association when using the adjusted data to compute the OR for the continuous BMI. Abbreviations: OR = odds ratio; BMI = body mass index; n = number of individuals; CI = confidence interval.

**Figure S4: Sensitivity analysis for transforming Asian-specific BMI reference criteria for the analysis of maternal BMI and post-term birth ≥41 weeks**

**Figure S4a) Association between maternal BMI and post-term birth using Asian-specific BMI criteria for Leung et al^34^**

Overall (I-squared = 94.0%, p = 0.000)

Stotland et al 2007 ^49^

Vaswani et al 2013 ^51^

Scott-Pilai et al 2013 ^47^

Schrauwers et al 2009 ^46^

Leung et al 2008 ^34^

Lumme et al 1995 ^35^

Arrowsmith et al 2011^19^

Manzanares et al 2012 ^37^

Nohr et al 2009 ^40^

Basu et al 2010 ^20^

Halloran et al 2012 ^27^

10878

1985

28548

348

27372

8719

19661

3938

3360

767

339126

2402

147

6212

3

4118

439

4979

279

203

132

51420

1.13 (1.05, 1.21)

1.30 (1.22, 1.40)

1.22 (1.04, 1.44)

0.96 (0.86, 1.07)

1.17 (0.53, 2.60)

1.10 (1.05, 1.16)

1.32 (1.19, 1.47)

1.24 (1.20, 1.28)

0.89 (0.78, 1.02)

1.09 (0.99, 1.20)

1.13 (0.96, 1.34)

1.05 (1.04, 1.05)

1

.2

.5

1.5

2

2.5

**Source**

**Participants**

**(n)**

**OR per 5 BMI units**

**(95% CI)**

**Post-term**

**≥41 weeks (n)**

**Figure S4b) Association between maternal BMI and post-term birth using General population BMI criteria for Leung et al^34^**

1.13 (1.05, 1.21)

1.30 (1.22, 1.40)

1.09 (0.99, 1.20)

1.13 (0.96, 1.34)

0.96 (0.86, 1.07)

1.32 (1.19, 1.47)

1.22 (1.04, 1.44)

1.17 (0.53, 2.60)

0.89 (0.78, 1.02)

1.05 (1.04, 1.05)

1.11 (1.05, 1.17)

1.24 (1.20, 1.28)

1

.2

.5

1

1.5

2

2.5

Overall (I-squared = 94.0%, p = 0.000)

Stotland et al 2007^54^

Vaswani et al 2013^58^

Scott-Pilai et al 2013^57^

Schrauwers et al 2009^60^

Leung et al 2008^32^

Lumme et al 1995^33^

Arrowsmith et al 2011^56^

Manzanares et al 2012^62^

Nohr et al 2009^68^

Basu et al 2010^59^

Halloran et al 2012^18^

10878

1985

28548

348

27372

8719

19661

3938

3360

767

339126

4118

439

4979

279

203

132

51420

**Source**

**Participants (n)**

**OR per 5 BMI units**

**(95% CI)**

**Post-term birth**

**≥41 weeks (n)**

2402

147

6212

3

Figure legend: Sensitivity analysis exploring the influence of using the Asian-specific BMI criteria for the study by Leung et al ^34^ on the overall effect size (OR) and 95% confidence interval for the association between maternal BMI and post-term birth ≥41 weeks. Abbreviations: OR = odds ratio; BMI = body mass index; n = number of individuals; CI = confidence interval.

**Table S6: Nonlinear meta-analyses using cubic splines regression**

**a) Post-term birth ≥42 weeks**

| Number of observations = 17 | | | | | |  |  |
| --- | --- | --- | --- | --- | --- | --- | --- |
|  |  | Coef. | Std. err. | z | P | 95% Confidence Interval | |
| Overall |  |  |  |  |  |  |  |
|  | spline1 | 0.0479 | 0.0096 | 5.00 | <0.001 | 0.0291 | 0.0667 |
|  | spline2 | -0.0302 | 0.0099 | -3.06 | 0.002 | -0.0495 | -0.0108 |

**b) Post-term birth ≥41 weeks**

| Number of observations = 11 | | | | | |  |  |
| --- | --- | --- | --- | --- | --- | --- | --- |
|  |  | Coef. | Std. err. | z | P | 95% Confidence Interval | |
| Overall |  |  |  |  |  |  |  |
|  | spline1 | 0.020952 | 0.007184 | 2.92 | 0.004 | 0.006873 | 0.0350315 |
|  | spline2 | -0.003860 | 0.003193 | -1.21 | 0.227 | -0.010119 | 0.0023986 |

**c) Post-term birth ≥41 weeks including Lumme et al^35^**

| Number of observations = 11 | | | | | |  |  |
| --- | --- | --- | --- | --- | --- | --- | --- |
|  |  | Coef. | Std. err. | z | P | 95% Confidence Interval | |
| Overall |  |  |  |  |  |  |  |
|  | spline1 | 0.03685 | 0.011754 | 3.14 | 0.002 | 0.013812 | 0.059888 |
|  | spline2 | -0.04136 | 0.022377 | -1.85 | 0.065 | -0.08521 | 0.002503 |

Footnote: Nonlinearity was assessed by testing that the coefficient of the second spline was equal to zero.

Abbreviations: z = value for the z statistic

Coef. = coefficient

Std. Err. = Standard error

P = p-value

**Table S7: Egger’s test for publication bias for post-term birth (≥ 42 weeks and ≥41 weeks)**

|  | **Std_Eff** | **Coef.** | **Std. Err.** | **t** | **P** | **[95% Conf. Interval]** | |
| --- | --- | --- | --- | --- | --- | --- | --- |
| **≥ 42 weeks** | slope | .1796733 | .0287235 | 6.26 | 0.000 | .119072 | .2402747 |
|  | bias | -1.150876 | 2.149826 | -0.54 | 0.599 | -5.686613 | 3.38486 |
|  | Test of H0: no small-study effects P = 0.599, Number of studies = 19, Root MSE = 7.443 | | | | | | |
| **≥ 41 weeks** | slope | .0439365 | .0175043 | 2.51 | 0.033 | .004339 | .083534 |
|  | bias | 2.066102 | 1.355701 | 1.52 | 0.162 | -1.000708 | 5.132911 |
|  | Test of H0: no small-study effects P = 0.162, Number of studies = 11, Root MSE = 3.768 | | | | | | |

Footnote: Egger's test for small-study effects: Regression of the standard normal deviate of intervention effect estimate against its standard error.

Abbreviations: Std_Eff = standard normal deviate of intervention effect

Coef. = coefficient

Std. Err. = Standard error

t = t-statistics

P = p-value

**Table S8: Maternal BMI and post-term birth ≥ 42 weeks sensitivity analysis**

|  | **Linear analyses**  OR  (95% CI) | **Nonlinear Analyses: BMI Midpoint (kg/m^2^)**  OR (95% CI) | | | | | | |
| --- | --- | --- | --- | --- | --- | --- | --- | --- |
| **Model** | **5 BMI units** | **17.5** | **22.5** | **27.5** | **32.5** | **37.5** | **42.5** | **47.5** |
| Arora et al 2013 ^18^ | 1.19 (1.12,1.27) | 0.80 (0.74,0.87) | 1 | 1.24 (1.15,1.34) | 1.42 (1.28,1.58) | 1.54 (1.37,1.73) | 1.62 (1.42,1.85) | 1.71 (1.46,1.99) |
| Bhattacharya et al 2007 ^21^ | 1.19 (1.11,1.26) | 0.81 (0.74,0.89) | 1 | 1.24 (1.14,1.34) | 1.41 (1.26,1.59) | 1.55 (1.36,1.76) | 1.65 (1.44,1.90) | 1.76 (1.50,2.06) |
| Cedergren et al 2004 ^24^ | 1.19 (1.11,1.28) | 0.80 (0.72,0.89) | 1 | 1.25 (1.14,1.38) | 1.44 (1.25,1.66) | 1.57 (1.34,1.85) | 1.67 (1.40,2.00) | 1.77 (1.44,2.17) |
| El-Gilani et al 2009 ^26^ | 1.17 (1.10,1.25) | 0.82 (0.73,0.89) | 1 | 1.23 (1.14,1.32) | 1.40 (1.26,1.56) | 1.53 (1.36,1.72) | 1.63 (1.45,1.84) | 1.74  (1.54,1.97) |
| Halloran et al 2012 ^27^ | 1.20 (1.14,1.27) | 0.79 (0.75,0.83) | 1 | 1.26 (1.21,1.32) | 1.45 (1.36,1.55) | 1.59  (1.45,1.75) | 1.70 (1.48,1.94) | 1.80 (1.48,1.94) |
| Johnson et al 1992 ^28^ | 1.19 (1.12,1.27) | 0.80 (0.74,0.88) | 1 | 1.24 (1.15,1.34) | 1.42 (1.27,1.58) | 1.53 (1.36,1.73) | 1.62 (1.42,1.85) | 1.70 (1.46,1.99) |
| Kashan et al 2009 ^29^ | 1.20 (1.12,1.28) | 0.81 (0.74,0.89) | 1 | 1.24 (1.14,1.34) | 1.43 (1.27,1.60) | 1.58 (1.38,1.80) | 1.71 (1.48,1.97) | 1.84 (1.56,2.17) |
| Kitiyodom et al 2008 ^31^ | 1.16 (1.10,1.24) | 0.83 (0.76,0.90) | 1 | 1.21 (1.12,1.31) | 1.37 (1.23,1.54) | 1.50 (1.32,1.69) | 1.59 (1.40,1.82) | 1.69 (1.47,1.96) |
| Konje et al 1993 ^33^ | 1.21 (1.14,1.28) | NA | NA | NA | NA | NA | NA | NA |
| Knight et al 2010 ^32^ | 1.19 (1.12,1.26) | 0.81 (0.74,0.87) | 1 | 1.24 (1.15,1.34) | 1.42 (1.27,1.58) | 1.55 (1.37,1.75) | 1.65 (1.44,1.89) | 1.75 (1.49,2.04) |
| Morgan et al 2014 ^38^ | 1.18 (1.12,1.26) | NA | NA | NA | NA | NA | NA | NA |
| Olesen et al 2006 ^41^ | 1.19 (1.12,1.27) | 0.80 (0.73,0.88) | 1 | 1.25 (1.15,1.35) | 1.43 (1.27,1.61) | 1.55 (1.36,1.78) | 1.65 (1.43,1.90) | 1.74 (1.48,2.05) |
| Raatikainen et al 2006 ^42^ | 1.19 (1.12,1.27) | 0.80 (0.74,0.88) | 1 | 1.24 (1.15,1.34) | 1.42 (1.27,1.60) | 1.55 (1.37,1.77) | 1.67 (1.44,1.90) | 1.76 (1.50,2.06) |
| Rode et al 2005 ^44^ | 1.19 (1.12,1.26) | 0.81 (0.74,0.89) | 1 | 1.23 (1.14,1.33) | 1.41 (1.26,1.58) | 1.54 (1.36,1.75) | 1.64 (1.43,1.89) | 1.75 (1.49,2.05) |
| Roos et al 2010 ^45^ | 1.17 (1.12,1.23) | 0.81 (0.74,0.89) | 1 | 1.23 (1.14,1.33) | 1.40 (1.25,1.57) | 1.51 (1.34,1.57) | 1.60 (1.38,1.85) | 1.68 (1.40,2.02) |
| Stotland et al 2007 ^49^ | 1.18 (1.11,1.25) | 0.81 (0.74,0.89) | 1 | 1.23 (1.14,1.33) | 1.40 (1.25,1.56) | 1.52 (1.34,1.72) | 1.61 (1.41,1.85) | 1.71 (1.46,1.99) |
| Vinturache et al 2014 ^52^ | 1.19 (1.12,1.26) | 0.80 (0.74,0.88) | 1 | 1.24 (1.15,1.34) | 1.42 (1.27,1.59) | 1.55 (1.37,1.76) | 1.65 (1.44,1.89) | 1.75 (1.50,2.05) |
| Voigt et al 2008 ^53^ | 1.20 (1.12,1.28) | 0.80 (0.73,0.87) | 1 | 1.25 (1.15,1.35) | 1.43 (1.28,1.60) | 1.57 (1.37,1.80) | 1.71 (1.45,2.02) | No data |
| Yazdani et al 2012 ^54^ | 1.19 (1.12,1.27) | 0.80 (0.74,0.87) | 1 | 1.25 (1.15,1.35) | 1.43 (1.28,1.60) | 1.56 (1.38,1.77) | 1.66 (1.45,1.91) | 1.76 (1.50,2.06) |

Footnote: Sensitivity analyses were performed by excluding one study at a time from the meta-analysis to identify the effect of any one individual study. The summary OR per 5 BMI units, obtained in the linear dose-response analysis, ranged from 1.16 (1.10,1.24) when the study by Kitiyodom et al^31^ was removed to 1.20 (1.12,1.28) when either of the studies by Kashan et al^29^ and Voigt et al^53^ were excluded. Consistently, for the nonlinear analysis the lowest ORs for all overweight and obese BMI midpoints resulted from the exclusion of the study by Kitiyodom et al^31^. Abbreviations: NA = not applicable as study was excluded from nonlinear analysis for reporting only 2 BMI categories.

**Table S9: Maternal BMI and post-term birth ≥ 41 weeks sensitivity analysis**

|  | **Linear analyses**  OR  (95% CI) | **Nonlinear Analyses: BMI Midpoint (kg/m^2^)**  OR (95% CI) | | | | | |
| --- | --- | --- | --- | --- | --- | --- | --- |
| **Model** | **5 BMI units** | **17.5** | **22.5** | **27.5** | **32.5** | **37.5** | **42.5** |
| Arrowsmith et al 2011 ^19^ | 1.10 (1.05,1.15) | 0.87 (0.77,0.97) | 1 | 1.08 (1.05,1.11) | 1.05 (0.99,1.11) | 0.94 (0.76,1.17) | 0.82 (0.54,1.25) |
| Basu et al 2010 ^20^ | 1.12 (1.05,1.18) | 0.84 (0.75,0.94) | 1 | 1.13 (1.07,1.19) | 1.15 (1.04,1.26) | 1.08 (0.87,1.34) | 0.99 (0.67,1.45) |
| Halloran et al 2012 ^27^ | 1.13 (1.04,1.22) | 0.82 (0.73,0.92) | 1 | 1.13 (1.07,1.20) | 1.13 (0.98,1.30) | 1.02 (0.73,1.41) | 0.87 (0.50,1.54) |
| Leung et al 2008 ^34^ | 1.12 (1.05,1.19) | 0.84 (0.75,0.95) | 1 | 1.13 (1.06,1.21) | 1.17 (1.07,1.28) | 1.12 (0.91,1.38) | 1.04 (0.71,1.53) |
| **Lumme et al 1995 ^35^** | **1.10 (1.04,1.16)** | **0.91 (0.85,0.97)** | **1** | **1.11 (1.04,1.20)** | **1.22 (1.07,1.39)** | **1.33 (1.10,1.59)** | **1.43 (1.13,1.83)** |
| Manzanares et al 2012 ^37^ | 1.14 (1.07,1.21) | 0.84 (0.76,0.94) | 1 | 1.14 (1.08,1.21) | 1.19 (1.09,1.31) | 1.17 (0.95,1.43) | 1.10 (0.76,1.60) |
| Nohr et al 2009 ^40^ | 1.12 (1.05,1.19) | 0.84 (0.75,0.93) | 1 | 1.13 (1.07,1.20) | 1.15 (1.05,1.27) | 1.09 (0.87,1.35) | 0.99 (0.66,1.46) |
| Schrauwers et al 2009 ^46^ | 1.12 (1.05,1.18) | 0.85 (0.76,0.94) | 1 | 1.12 (1.07,1.19) | 1.15 (1.05,1.27) | 1.11 (0.90,1.36) | 1.02 (0.71,1.49) |
| Scott-Pilai et al 2013 ^47^ | 1.13 (1.05,1.22) | 0.83 (0.74,0.94) | 1 | 1.14 (1.06,1.22) | 1.16 (1.01,1.32) | 1.08 (0.83,1.43) | 0.98 (0.61,1.57) |
| Stotland et al 2007 ^49^ | 1.10 (1.04,1.17) | 0.86 (0.76,0.96) | 1 | 1.11 (1.07,1.16) | 1.14 (1.07,1.22) | 1.09 (0.89,1.34) | 1.01 (0.69,1.50) |
| Vaswani et al 2013 ^51^ | 1.11 (1.05,1.18) | 0.84 (0.76,0.94) | 1 | 1.12 (1.06,1.18) | 1.14 (1.04,1.25) | 1.08 (0.87,1.34) | 0.98 (0.66,1.45) |

Footnote: Sensitivity analyses were performed by excluding one study at a time from the meta-analysis to identify the effect of any one individual study. The summary OR per 5 BMI units, obtained in the linear dose-response analysis, ranged from 1.10 (1.04,1.16) when the study by Lumme et al^35^ was excluded to 1.13 (1.05,1.22) when the study by Scott-Pilai et al^47^ was excluded. In the nonlinear analysis, the biggest change on the ORs for all BMI midpoints occurr when excluding the study by Lumme et al^35^ resulting in a linear association between maternal BMI and prolonged pregnancy (fig. S5).

**Figure S5: Nonlinear dose-response analysis for maternal BMI and post-term birth ≥41 weeks, including all studies**

1.00

2.00

4.00

8.00

15

20

25

30

35

40

45

50

Nonlinear Model

95% CI

OR of post-term birth ≥41 weeks

Maternal BMI, kg/m^2^

Legend: Nonlinear meta-analysis for post-term ≥41 weeks when including Lumme et al^35^ study data. Despite the nonlinear appearance of the graph, linearity is not rejected (p = 0.065, table S6).

**Table S10: Meta-regression and sub-group results for post-term birth ≥ 42 weeks**

| **Variable** |  | **Effect and Significance** | | | **Heterogeneity Results** | | |
| --- | --- | --- | --- | --- | --- | --- | --- |
| Sub-group | **n studies** | **OR** | **Lower 95% CI** | **Upper 95% CI** | **Sub-group specific *I^2^* (%)** | **p value** | **Meta-regression *I^2^* (%)** |
| **All studies** | 19 | 1.18 | 1.11 | 1.26 |  | <0.001 | 98.1 |
| **Clinical Factors** | | | | | | | |
| **Assessment of BMI** |  |  |  |  |  |  | 96.12 |
| Self-reported | 9 | 1.143 | 1.075 | 1.215 | 91.3 | <0.001 |  |
| Measured | 10 | 1.206 | 1.122 | 1.297 | 97.5 | <0.001 |  |
| Unclear | 0 |  |  |  |  |  |  |
| **Assessment of Gestational Age** |  |  |  |  |  |  | 98.2 |
| Self-reported (LMP) | 2 | 1.377 | 0.964 | 1.967 | 88.2 | 0.004 |  |
| Measured (USS) | 12 | 1.184 | 1.095 | 1.281 | 98.8 | <0.001 |  |
| Unclear | 5 | 1.09 | 1.068 | 1.113 | 0 | 0.76 |  |
| **Induction of Labour or Caesarean Section - adjusted** |  |  |  |  |  |  | 94.63 |
| Yes | 3 | 1.301 | 1.289 | 1.313 | 0 | 0.651 |  |
| No | 16 | 1.155 | 1.095 | 1.218 | 95.4 | <0.001 |  |
| **Parity - adjusted** |  |  |  |  |  |  | 93.79 |
| Yes | 4 | 1.237 | 1.154 | 1.325 | 98.3 | <0.001 |  |
| No | 13 | 1.129 | 1.071 | 1.191 | 87.4 | <0.001 |  |
| Primiparous only | 2 | 1.214 | 1.134 | 1.3 | 0 | 0.472 |  |
| **Gestational diabetes - adjusted** |  |  |  |  |  |  | 96.85 |
| Yes | 4 | 1.196 | 0.952 | 1.503 | 87 | <0.001 |  |
| No | 14 | 1.188 | 1.12 | 1.259 | 97.4 | <0.001 |  |
| Unclear | 1 | 1.011 | 0.655 | 1.56 |  |  |  |
| **Hypertension / Pre-eclampsia - adjusted** |  |  |  |  |  |  | 98.31 |
| Yes | 3 | 1.385 | 1.131 | 1.696 | 56.4 | 0.101 |  |
| No | 15 | 1.161 | 1.086 | 1.24 | 98.6 | <0.001 |  |
| Unclear | 1 | 1.011 | 0.655 | 1.56 |  |  |  |
| **Methodology/context of included studies** | | | | | | | |
| **Geographic location** |  |  |  |  |  |  | 96.79 |
| Europe | 11 | 1.148 | 1.079 | 1.222 | 97.9 | <0.001 |  |
| North America | 4 | 1.158 | 0.972 | 1.378 | 87.4 | <0.001 |  |
| Asia | 4 | 1.42 | 1.066 | 1.892 | 73.1 | 0.011 |  |
| **Study Quality score** |  |  |  |  |  |  | 94.87 |
| 0-2 | 2 | 1.087 | 1.065 | 1.11 | 0 | 0.89 |  |
| 3-5 | 15 | 1.168 | 1.098 | 1.242 | 95.7 | <0.001 |  |
| 6-8 | 2 | 1.301 | 1.289 | 1.313 | 0 | 0.528 |  |
| **Study dimension** |  |  |  |  |  |  | 95.97 |
| Local | 6 | 1.19 | 0.988 | 1.432 | 86.5 | <0.001 |  |
| Regional | 8 | 1.133 | 1.059 | 1.212 | 86.4 | <0.001 |  |
| National | 5 | 1.181 | 1.093 | 1.275 | 98.8 | <0.001 |  |
| **Study start (per decade)** |  |  |  |  |  |  | 96.29 |
| 1970 | 1 | 1.219 | 1.138 | 1.307 |  |  |  |
| 1980 | 3 | 1.018 | 0.813 | 1.274 | 84.3 | 0.002 |  |
| 1990 | 6 | 1.203 | 1.125 | 1.287 | 98.5 | <0.001 |  |
| 2000 | 8 | 1.17 | 1.074 | 1.275 | 86.8 | <0.001 |  |
| 2010 | 1 | 1.011 | 0.655 | 1.56 |  |  |  |
| **Type of study** |  |  |  |  |  |  | 98.22 |
| Retrospective | 13 | 1.185 | 1.108 | 1.267 | 98.8 | <0.001 |  |
| Prospective | 6 | 1.17 | 0.882 | 1.552 | 79.9 | <0.001 |  |
| **Sample Size** |  |  |  |  |  |  | 98.33 |
| <1000 | 10 | 1.216 | 1.019 | 1.452 | 79.3 | <0.001 |  |
| 1000-<10000 | 4 | 1.204 | 1.145 | 1.267 | 48.7 | 0.119 |  |
| >=10000 | 5 | 1.122 | 1.016 | 1.239 | 99.6 | <0.001 |  |
| **Number of cases** |  |  |  |  |  |  | 98.33 |
| <100 | 7 | 1.124 | 0.818 | 1.545 | 76.4 | <0.001 |  |
| 100-<1000 | 4 | 1.344 | 1.147 | 1.575 | 83 | 0.001 |  |
| 1000-<10000 | 4 | 1.142 | 1.05 | 1.241 | 92.5 | <0.001 |  |
| >10000 | 4 | 1.144 | 1.024 | 1.277 | 99.6 | <0.001 |  |
| **Number of Exposure Categories** |  |  |  |  |  |  | 75.95 |
| 2 | 2 | 1.095 | 0.373 | 3.213 | 86.9 | 0.006 |  |
| 3 | 3 | 1.09 | 1.068 | 1.113 | 0 | 0.421 |  |
| 4 | 4 | 1.282 | 1.211 | 1.358 | 29.9 | 0.233 |  |
| 5 | 5 | 1.217 | 1.152 | 1.284 | 80.5 | <0.001 |  |
| 6 | 5 | 1.092 | 1.027 | 1.16 | 84.9 | <0.001 |  |
| **Methodology of this systematic review** | | | | | | | |
| **Publication Decade** |  |  |  |  |  |  | 98.18 |
| 1990s | 2 | 0.896 | 0.515 | 1.559 | 91.4 | 0.001 |  |
| 2000s | 10 | 1.199 | 1.14 | 1.262 | 93.7 | <0.001 |  |
| 2010s | 7 | 1.145 | 0.959 | 1.366 | 99.2 | <0.001 |  |
| **Study Identification** |  |  |  |  |  |  | 98.18 |
| Citation Search | 4 | 1.25 | 0.828 | 1.888 | 74.1 | 0.009 |  |
| Database Search | 12 | 1.2 | 1.115 | 1.291 | 98.8 | <0.001 |  |
| Reference List Search | 3 | 1.029 | 0.885 | 1.196 | 83.5 | 0.002 |  |
| **Adjustment of the odds ratios** |  |  |  |  |  |  | 93.8 |
| Unadjusted | 15 | 1.141 | 1.084 | 1.202 | 87.6 | <0.001 |  |
| Adjusted | 4 | 1.237 | 1.154 | 1.325 | 98.3 | <0.001 |  |

Footnote: Highlighted data show the results of the sub-group meta-analyses which resulted in a lack of statistically significant heterogeneity (*I^2^*<75%, p>0.05) when three or more studies were included in the meta-analysis. Abbreviations*: n studies* = number of studies, *OR* = odds ratio, *Lower 95% CI* = lower limit of the 95% confidence interval, *Upper 95% CI* = upper limit of the 95% confidence interval, LMP = last menstrual period, USS = ultrasound scan

**Table S11: Meta-regression and sub-group results for post-term birth ≥ 41 weeks**

| **Variable** |  | **Effect and Significance** | | | **Heterogeneity Results** | | |
| --- | --- | --- | --- | --- | --- | --- | --- |
| Sub-group | **n studies** | **OR** | **Lower 95% CI** | **Upper 95% CI** | **Sub-group specific *I^2^* (%)** | **p value** | **Meta-regression *I^2^* (%)** |
| **All studies** | 11 | 1.13 | 1.06 | 1.21 |  | <0.001 | 94 |
| **Clinical Factors** | | | | | | | |
| **Assessment of BMI** |  |  |  |  |  |  | 89.54 |
| Self-reported | 3 | 1.14 | 0.987 | 1.317 | 94.8 | <0.001 |  |
| Measured | 8 | 1.119 | 1.022 | 1.225 | 86.2 | <0.001 |  |
| Not available | 0 |  |  |  |  |  |  |
| **Assessment of Gestational Age** |  |  |  |  |  |  | 94.3 |
| Self-reported (LMP) | 0 |  |  |  |  |  |  |
| Measured (USS) | 4 | 1.176 | 1.036 | 1.335 | 97.8 | <0.001 |  |
| Not available | 7 | 1.09 | 0.989 | 1.201 | 79.6 | <0.001 |  |
| **Induction of Labour or Caesarean Section - adjusted** |  |  |  |  |  |  | 83.51 |
| Yes | 3 | 1.166 | 1.025 | 1.328 | 91.6 | <0.001 |  |
| No | 8 | 1.103 | 1.033 | 1.178 | 78.9 | <0.001 |  |
| **Parity - adjusted** |  |  |  |  |  |  | 83.51 |
| Yes | 3 | 1.166 | 1.025 | 1.328 | 91.6 | <0.001 |  |
| No | 8 | 1.103 | 1.033 | 1.178 | 78.9 | <0.001 |  |
| Primiparous only | 0 |  |  |  |  |  |  |
| **Gestational diabetes - adjusted** |  |  |  |  |  |  | 94.86 |
| Yes | 5 | 1.145 | 1.024 | 1.281 | 97.2 | <0.001 |  |
| No | 5 | 1.104 | 0.947 | 1.287 | 81.2 | <0.001 |  |
| Unclear | 1 | 1.105 | 1.046 | 1.167 |  |  |  |
| **Hypertension / Pre-eclampsia - adjusted** |  |  |  |  |  |  | 83.21 |
| Yes | 4 | 1.178 | 1.057 | 1.313 | 87.3 | <0.001 |  |
| No | 6 | 1.089 | 0.989 | 1.199 | 80.7 | <0.001 |  |
| Unclear | 1 | 1.105 | 1.046 | 1.167 |  |  |  |
| **Methodology/context of included studies** | | | | | | | |
| **Geographic location** |  |  |  |  |  |  | 92.77 |
| Europe | 5 | 1.095 | 0.962 | 1.246 | 91.5 | <0.001 |  |
| North America | 2 | 1.164 | 0.938 | 1.444 | 97.4 | <0.001 |  |
| Asia | 2 | 1.129 | 1.041 | 1.224 | 27 | 0.242 |  |
| Africa | 1 | 1.131 | 0.959 | 1.335 |  |  |  |
| Australia | 1 | 1.172 | 0.528 | 2.602 |  |  |  |
| **Study Quality score** |  |  |  |  |  |  | 85.32 |
| 0-2 | 1 | 1.172 | 0.528 | 2.602 |  |  |  |
| 3-5 | 7 | 1.103 | 1.032 | 1.179 | 81.9 | <0.001 |  |
| 6-8 | 3 | 1.166 | 1.025 | 1.328 | 91.6 | <0.001 |  |
| **Study dimension** |  |  |  |  |  |  | 87.05 |
| Local | 7 | 1.151 | 1.058 | 1.252 | 84.1 | <0.001 |  |
| Regional | 2 | 1.169 | 0.928 | 1.474 | 94.6 | <0.001 |  |
| National | 1 | 1.093 | 0.995 | 1.2 |  |  |  |
| **Study start (per decade)** |  |  |  |  |  |  | 94.18 |
| 1980 | 1 | 1.324 | 1.189 | 1.475 |  |  |  |
| 1990 | 3 | 1.164 | 1.039 | 1.305 | 87 | <0.001 |  |
| 2000 | 6 | 1.054 | 0.948 | 1.172 | 95.5 | <0.001 |  |
| 2010 | 1 | 1.223 | 1.041 | 1.436 |  |  |  |
| **Type of study** |  |  |  |  |  |  | 94.05 |
| Retrospective | 9 | 1.108 | 1.022 | 1.202 | 94.7 | <0.001 |  |
| Prospective | 2 | 1.201 | 0.994 | 1.45 | 85.7 | 0.008 |  |
| **Sample Size** |  |  |  |  |  |  | 94.87 |
| <1000 | 2 | 1.133 | 0.963 | 1.333 | 0 | 0.932 |  |
| 1000-<10000 | 4 | 1.121 | 0.952 | 1.32 | 86.6 | <0.001 |  |
| >=10000 | 5 | 1.127 | 1.021 | 1.244 | 97.1 | <0.001 |  |
| **Number of cases** |  |  |  |  |  |  | 87.19 |
| <100 | 1 | 1.172 | 0.528 | 2.602 |  |  |  |
| 100-<1000 | 5 | 1.123 | 0.982 | 1.284 | 82.1 | <0.001 |  |
| 1000-<10000 | 4 | 1.152 | 1.043 | 1.272 | 91.3 | <0.001 |  |
| >10000 | 1 | 1.046 | 1.037 | 1.054 |  |  |  |
| **Number of Exposure Categories** |  |  |  |  |  |  | 92.68 |
| 4 | 4 | 1.288 | 1.219 | 1.361 | 0 | 0.428 |  |
| 5 | 2 | 1.129 | 1.041 | 1.224 | 27 | 0.242 |  |
| 6 | 5 | 1.049 | 0.948 | 1.162 | 96.4 | <0.001 |  |
| **Methodology of this systematic review** | | | | | | | |
| **Publication Decade** |  |  |  |  |  |  | 93.51 |
| 1990s | 1 | 1.324 | 1.189 | 1.475 |  |  |  |
| 2000s | 4 | 1.165 | 1.045 | 1.298 | 80.5 | 0.002 |  |
| 2010s | 6 | 1.074 | 0.972 | 1.186 | 95.6 | <0.001 |  |
| **Study Identification** |  |  |  |  |  |  | 94.23 |
| Citation Search | 1 | 0.893 | 0.784 | 1.017 |  |  |  |
| Database Search | 8 | 1.132 | 1.046 | 1.224 | 95.2 | <0.001 |  |
| Reference List Search | 2 | 1.322 | 1.188 | 1.47 | 0 | 0.766 |  |
| **Adjustment of the odds ratios** |  |  |  |  |  |  | 82.42 |
| Unadjusted | 7 | 1.091 | 1.020 | 1.168 | 79.8 | <0.001 |  |
| Adjusted | 4 | 1.178 | 1.057 | 1.313 | 87.3 | <0.001 |  |

Footnote: Highlighted data show the results of the sub-group meta-analyses which resulted in a lack of statistically significant heterogeneity (*I^2^*<75%, p>0.05) when three or more studies were included in the meta-analysis. Abbreviations*: n studies* = number of studies, *OR* = odds ratio, *Lower 95% CI* = lower limit of the 95% confidence interval, *Upper 95% CI* = upper limit of the 95% confidence interval, LMP = last menstrual period, USS = ultrasound scan

**Supporting Information References**

1. Wells GA, Shea B, O’connell D, et al. The Newcastle-Ottawa Scale (NOS) for assessing the quality of nonrandomised studies in meta-analyses. 2000.

2. Bogaerts A, Witters I, Van den Bergh BRH, Jans G, Devlieger R. Obesity in pregnancy: Altered onset and progression of labour. *Midwifery.* 2013;29:1303-1313.

3. Castro LC, Avina RL. Maternal obesity and pregnancy outcomes. *Current Opinion in Obstetrics and Gynecology.* 2002;14(6):601-606.

4. Catalano PM, Ehrenberg HM. The short- and long-term implications of maternal obesity on the mother and her offspring. *BJOG: Int J Obstet Gynaecol.* 2006;113.

5. Caughey AB, Snegovskikh VV, Norwitz ER. Postterm pregnancy: how can we improve outcomes? *Obstetrical & gynecological survey.* 2008;63(11):715-724.

6. Gülmezoglu AM, Crowther CA, Middleton P, Heatley E. Induction of labour for improving birth outcomes for women at or beyond term. *Cochrane Database Syst Rev.* 2012;6(6).

7. Heslehurst N, Simpson H, Ells LJ, et al. The impact of maternal BMI status on pregnancy outcomes with immediate short-term obstetric resource implications: a meta-analysis. *Obesity Reviews.* 2008;9(6):635-683.

8. Linne Y. Effects of Obesity on Women's Reproduction and Complications During Pregnancy. *Obesity Reviews.* 2004;5:137-143.

9. Lutsiv O, Mah J, Beyene J, McDonald SD. The effects of morbid obesity on maternal and neonatal health outcomes: a systematic review and meta‐analyses. *Obesity Reviews.* 2015;16(7):531-546.

10. McDonald SD, Han Z, Mulla S, Beyene J, on behalf of the Knowledge Synthesis Group. Overweight and obesity in mothers and risk of preterm birth and low birth weight infants: systematic review and meta-analyses. *British Medical Journal.* 2010;341:c3428.

11. Nuthalapaty FS, Rouse DJ. The impact of obesity on obstetrical practice and outcome. *Clinical obstetrics and gynecology.* 2004;47(4):898-913.

12. Torloni MR, Betran AP, Daher S, et al. Maternal BMI and preterm birth: A systematic review of the literature with meta-analysis. *The Journal of Maternal-Fetal and Neonatal Medicine.* 2009;22(11):957-970.

13. Vasudevan C, Renfrew M, McGuire W. Fetal and perinatal consequences of maternal obesity. *Archives of Disease in Childhood. Fetal and Neonatal Edition.* 2011;96(5).

14. Walker N, Gan JH. Prolonged pregnancy. *Obstetrics, Gynaecology and Reproductive Medicine.* 01 Mar 2015;25(3):83-87.

15. Wolfe H. High prepregnancy body-mass index - a maternal-fetal risk factor. *New England Journal of Medicine.* 1998;338(3):191.

16. Abenhaim HA, Kinch RA, Morin L, Benjamin A, Usher R. Effect of prepregnancy body mass index categories on obstetrical and neonatal outcomes. *Archives of Gynecology & Obstetrics.* 2007;275(1):39-43.

17. Al-Rayyan E, Shwayat R, AL-Sumadi A, et al. The Effect of High Maternal Body Mass Index before Pregnancy on Pregnancy Outcome. *Journal of the Royal Medical Services.* 2010;17(2):11-15.

18. Arora R, Arora D, Patumanond J. Adverse pregnancy outcomes in women with high pre-pregnancy body mass index. *Open Journal of Obstetrics and Gynecology.* 2013;3:285-291.

19. Arrowsmith S, Wray S, Quenby S. Maternal obesity and labour complications following induction of labour in prolonged pregnancy. *BJOG: An International Journal of Obstetrics and Gynaecology.* April 2011;118(5):578-588.

20. Basu JK, Jeketera CM, Basu D. Obesity and its outcomes among pregnant South African women. *International Journal of Gynecology and Obstetrics.* August 2010;110(2):101-104.

21. Bhattacharya S, Campbell DM, Liston WA, Bhattacharya S. Effect of Body Mass Index on pregnancy outcomes in nulliparous women delivering singleton babies. *BMC Public Health.* 2007;7:168.

22. Briese V, Voigt M, Wisser J, Borchardt U, S. S. Risks of pregnancy and birth in obese primiparous women: an analysis of German perinatal statistics. *Archives of Gynecology and Obstetrics.* 2011;283(2):249-253.

23. Caughey AB, Stotland NE, Washington AE, Escobar GJ. Who is at risk for prolonged and postterm pregnancy? *American Journal of Obstetrics and Gynecology.* June 2009;200(6):683.e681-683.e685.

24. Cedergren MI. Maternal morbid obesity and the risk of adverse pregnancy outcome. *Obstetrics & Gynecology.* 2004;103(2):219-224.

25. Denison FC, Price J, Graham C, Wild S, Liston WA. Maternal obesity, length of gestation, risk of postdates pregnancy and spontaneous onset of labour at term. *BJOG: An International Journal of Obstetrics and Gynaecology.* May 2008;115(6):720-725.

26. El-Gilany AH, Hammad S. Body mass index and obstetric outcomes in pregnant in Saudi Arabia: A prospective cohort study. *Annals of Saudi Medicine.* September-October 2010;30(5):376-380+421.

27. Halloran DR, Cheng YW, Wall TC, MacOnes GA, Caughey AB. Effect of maternal weight on postterm delivery. *Journal of Perinatology.* February 2012;32(2):85-90.

28. Johnson JW, Longmate JA, Frentzen B. Excessive maternal weight and pregnancy outcome. *American Journal of Obstetrics & Gynecology.* 1992;167(2):353-370; discussion 370-352.

29. Khashan AS, Kenny LC. The effects of maternal body mass index on pregnancy outcome. *European Journal of Epidemiology.* November 2009;24(11):697-705.

30. Kistka ZAF, Palomar L, Boslaugh SE, DeBaun MR, DeFranco EA, Muglia LJ. Risk for postterm delivery after previous postterm delivery. *American Journal of Obstetrics and Gynecology.* March 2007;196(3):241.e241-241.e246.

31. Kitiyodom S, Tongswatwong P. Pregnancy Outcomes of Parturients with Excessive-weight in Maharat Nakorn Ratchasima Hospital. *Thai Journal of Obstetrics and Gynaecology.* 2008;16 214-220.

32. Knight M, Kurinczuk JJ, Spark P, Brocklehurst P, System UKOS. Extreme obesity in pregnancy in the United Kingdom. *Obstetrics & Gynecology.* 2010;115(5):989-997.

33. Konje JC, Imrie A, Hay DM. Pregnancy in Obese Women. *Journal of Obstetrics and Gynaecology.* 1993;13:413-418.

34. Leung TY, Leung TN, Sahota DS, et al. Trends in maternal obesity and associated risks of adverse pregnancy outcomes in a population of Chinese women. *BJOG: An International Journal of Obstetrics & Gynaecology.* 2008;115(12):1529-1537.

35. Lumme R, Rantakallio P, Hartikainen AL, Jarvelin MR. Pre-pregnancy weight and it's relation to pregnancy outcome. *Obstetrics and Gynaecology.* 1995;15:69-75.

36. Mancuso A, D'Anna R, Leonardi R. Pregnancy in the obese patient. *European Journal of Obstetrics and Gynecology and Reproductive Biology.* 1991;39:83-86.

37. Sebastián Manzanares G, Ángel Santalla H, Irene Vico Z, López Criado MS, Alicia Pineda L, José Luis Gallo V. Abnormal maternal body mass index and obstetric and neonatal outcome. *The Journal of Maternal-Fetal & Neonatal Medicine.* 2012/03/01 2012;25(3):308-312.

38. Morgan KL, Rahman MA, Hill RA, et al. Physical Activity and Excess Weight in Pregnancy Have Independent and Unique Effects on Delivery and Perinatal Outcomes. *PLoS ONE.* 2014;9(4):e94532.

39. Navid S, Arshad S, Atif K, Arshad Meo R. Impact of high maternal body mass index on length of gestation and maternal delivery outcomes. *Rawal Medical Journal.* 2013;38(3):279-282.

40. Nohr EA, Timpson NJ, Andersen CS, Smith GD, Olsen J, Sorensen TIA. Severe obesity in young women and reproductive health: The danish national birth cohort. *PLoS ONE.* 2009;4(12).

41. Olesen AW, Westergaard JG, Olsen J. Prenatal risk indicators of a prolonged pregnancy. The Danish Birth Cohort 1998-2001. *Acta obstetricia et gynecologica Scandinavica.* 2006;85(11):1338-1341.

42. Raatikainen K, Heiskanen N, Heinonen S. Transition from Overweight to Obesity Worsens Pregnancy Outcome in a BMI-dependent Manner. *Obesity* 2006;14(1):165-171.

43. Robinson HE, O'Connell CM, Joseph KS, McLeod NL. Maternal outcomes in pregnancies complicated by obesity. *Obstetrics & Gynecology.* 2005;106(6):1357-1364.

44. Rode L, Nilas L, Wojdemann K, Tabor A. Obesity-related complications in Danish single cephalic term pregnancies. *Obstetrics & Gynecology.* 2005;105(3):537-542.

45. Roos N, Sahlin L, Ekman-Ordeberg G, Kieler H, Stephansson O. Maternal risk factors for postterm pregnancy and cesarean delivery following labor induction. *Acta Obstetricia et Gynecologica Scandinavica.* August 2010;89(8):1003-1010.

46. Schrauwers C, Dekker G. Maternal and perinatal outcome in obese pregnant patients. *Journal of Maternal-Fetal & Neonatal Medicine.* 2009;22(3):218-226.

47. Scott-Pillai R, Spence D, Cardwell CR, Hunter A, Holmes VA. The impact of body mass index on maternal and neonatal outcomes: A retrospective study in a UK obstetric population, 2004-2011. *BJOG: An International Journal of Obstetrics and Gynaecology.* July 2013;120(8):932-939.

48. Sharief M, Tarik A. Obesity in pregnancy. *Qatar Medical Journal.* 2000;9(2):48-50.

49. Stotland NE, Washington AE, Caughey AB. Prepregnancy body mass index and the length of gestation at term. *American Journal of Obstetrics and Gynecology.* October 2007;197(4):378.e371-378.e375.

50. Usha Kiran TS, Hemmadi S, Bethel J, Evans J. Outcome of pregnancy in a woman with an increased body mass index. *BJOG: An International Journal of Obstetrics & Gynaecology.* 2005;112(6):768-772.

51. Vaswani PR, Balachandran L. Pregnancy outcomes in a population with high prevalence of obesity: How bad is it? *Clinical Epidemiology and Global Health.* April 2013;1(1):5-11.

52. Vinturache A, Moledina N, McDonald S, Slater D, Tough S. Pre-pregnancy Body Mass Index (BMI) and delivery outcomes in a Canadian population. *BMC Pregnancy and Childbirth.* 2014;14(1):1-10.

53. Voigt M, Zygmunt M, Henrich W, Straube S, Carstensen M, V. B. Analysis of subgroup of pregnant women in Germany. *Geburtsh Frauenheilk. 16th communication: morbid obesity: pregnancy risks, birth risks and status of the newborn.* 2008;68:794-800.

54. Yazdani S, Yosofniyapasha Y, Nasab BH, Mojaveri MH, Bouzari Z. Effect of maternal body mass index on pregnancy outcome and newborn weight. *BMC Research Notes.* 2012;5(1):1-4.
